# Tumor-derived exosomal miR-934 induces macrophage M2 polarization to promote liver metastasis of colorectal cancer

**DOI:** 10.1186/s13045-020-00991-2

**Published:** 2020-11-19

**Authors:** Senlin Zhao, Yushuai Mi, Bingjie Guan, Binbin Zheng, Ping Wei, Yanzi Gu, Zhengxiang Zhang, Sanjun Cai, Ye Xu, Xinxiang Li, Xuefeng He, Xinyang Zhong, Guichao Li, Zhiyu Chen, Dawei Li

**Affiliations:** 1 Department of Colorectal Surgery, Fudan University Shanghai Cancer Center, 270 Dong’an Road, Shanghai, 200032 China; 2 Department of Oncology, Shanghai Medical College, Fudan University, 270 Dong’an Road, Shanghai, 200032 China; 3 Department of Gastrointestinal Surgery, The Second Hospital, Cheeloo College of Medicine, Shandong University, No. 247 Beiyuan Street, Jinan, 250033 China; 4 Department of General Surgery, Shanghai General Hospital, School of Medicine, Shanghai Jiaotong University, 85 Wujin Road, Shanghai, 200080 China; 5 Cancer Institute, Fudan University Shanghai Cancer Center, 270 Dong’an Road, Shanghai, 200032 China; 6 Department of Pathology, Fudan University Shanghai Cancer Center, 270 Dong’an Road, Shanghai, 200032 China; 7 Department of Biobank, Fudan University Shanghai Cancer Center, 270 Dong’an Road, Shanghai, 200032 China; 8 Department of Oncology, Yijishan Hospital of Wannan Medical College, No. 2 Zheshan Road, Wuhu, 241001 Anhui China; 9 Department of Radiation Oncology, Fudan University Shanghai Cancer Center, 270 Dong’an Road, Shanghai, 200032 China; 10 Department of Medical Oncology, Fudan University Shanghai Cancer Center, 270 Dong’an Road, Shanghai, 200032 China

**Keywords:** Colorectal cancer liver metastasis, Exosome, miR-934, M2 macrophage polarization, Premetastatic niche

## Abstract

**Background:**

Mounting evidence has demonstrated the vital importance of tumor-associated macrophages (TAMs) and exosomes in the formation of the premetastatic niche. However, the molecular mechanisms by which tumor-derived exosomal miRNAs interact with TAMs underlying premetastatic niche formation and colorectal cancer liver metastasis (CRLM) remain largely unknown.

**Methods:**

Transmission electron microscopy and differential ultracentrifugation were used to verify the existence of exosomes. In vivo and in vitro assays were used to identify roles of exosomal miR-934. RNA pull-down assay, dual-luciferase reporter assay, etc. were applied to clarify the mechanism of exosomal miR-934 regulated the crosstalk between CRC cells and M2 macrophages.

**Results:**

In the present study, we first demonstrated the aberrant overexpression of miR-934 in colorectal cancer (CRC), especially in CRLM, and its correlation with the poor prognosis of CRC patients. Then, we verified that CRC cell-derived exosomal miR-934 induced M2 macrophage polarization by downregulating PTEN expression and activating the PI3K/AKT signaling pathway. Moreover, we revealed that hnRNPA2B1 mediated miR-934 packaging into exosomes of CRC cells and then transferred exosomal miR-934 into macrophages. Interestingly, polarized M2 macrophages could induce premetastatic niche formation and promote CRLM by secreting CXCL13, which activated a CXCL13/CXCR5/NFκB/p65/miR-934 positive feedback loop in CRC cells.

**Conclusions:**

These findings indicate that tumor-derived exosomal miR-934 can promote CRLM by regulating the crosstalk between CRC cells and TAMs. These findings reveal a tumor and TAM interaction in the metastatic microenvironment mediated by tumor-derived exosomes that affects CRLM. The present study also provides a theoretical basis for secondary liver cancer.

## Introduction

Although the combination of surgery, chemotherapy, targeted therapy, and immunotherapy has partially improved the clinical efficacy of colorectal cancer (CRC) therapy, CRC still ranks third in terms of incidence and is the second leading cause of cancer-related deaths worldwide [1, 2]. Approximately 50% of cancer-related deaths arise from colorectal cancer liver metastasis (CRLM) [3]. CRLM is a multistep biological process regulated by key genes that mediate crosstalk between tumor cells and the tumor microenvironment (TME) [4]. Accordingly, exploring pivotal metastasis‐driven genes and their underlying mechanisms as well as identifying effective therapeutic targets to block metastasis may aid in the development of effective treatment strategies to improve the prognosis of patients with CRLM.

It has been reported that primary tumors create a favorable TME in secondary organs and tissue sites for subsequent metastases, which could be primed and established through a complex interplay of primary tumor-derived factors, tumor-mobilized bone marrow-derived cells, and local stromal components [5]. It is widely believed that cells from the TME contribute to tumor metastasis [6, 7]. Accumulating evidence suggests that exosomes play key roles in remodeling the TME and tumor metastasis by transferring signal peptides, noncoding RNAs, or DNA to neighboring cells or tissues [8, 9]. MicroRNAs (miRNAs), which are present in exosomes, can be taken up by neighboring or distant cells, and they subsequently modulate the recipient cells [10]. As a major class of 17–24 nt small noncoding RNAs, miRNAs regulate the expression of target messenger RNAs (mRNAs) by binding to their 3′‐untranslated regions (3′‐UTRs), thereby contributing to the progression of various cancers [11]. The dysregulation of exosomal miRNAs can influence the crosstalk between cancer cells and the TME [12]. Macrophages are the most abundant infiltrative immune-related stromal cells present in and around tumors, and they can be polarized into classically activated M1 macrophages or alternatively activated M2 macrophages by different stimuli [13]. Tumor-associated macrophages (TAMs), which are considered to be M2-like, exist in the TME and influence the metastasis of various cancers by interacting with cancer cells [14]. The crosstalk between cancer cells and M2 macrophages has been investigated extensively; however, the mechanisms underlying the activation of M2 macrophages by tumor cells remain unclear in colorectal cancer and are even more obscure in CRLM.

In the present study, we investigated the expression pattern of miR-934 in CRLM and evaluated whether CRC-derived exosomes could induce M2 macrophage polarization via secretion of miR-934, hoping that our findings may aid in identifying a novel biomarker specific for CRLM and in the development of new strategies for predicting the risk of CRLM.

## Materials and methods

### Clinical specimens and ethical approval

Human CRC and adjacent normal mucosa samples were obtained from CRC patients who underwent surgery at Fudan University Shanghai Cancer Center between January 2008 and September 2009. Prior to tumor resection, serum was extracted from blood samples of CRC patients by centrifugation of blood at 3000 g for 10 min. All clinicopathological diagnoses were confirmed by at least two pathologists according to the guidelines of the American Joint Committee on Cancer (AJCC). The present study was approved by the Ethics Committee of Fudan University Shanghai Cancer Center (FUSCC, ID: 050432-4-1911D), and informed consent was obtained from all patients before enrollment in this study.

### Statistical analysis

All in vitro experiments were performed in triplicate, and their results are presented as the mean ± SEM (parametric data) or the median and range (nonparametric data). The association between miR-934 expression and the clinicopathological variables of CRC patients was statistically verified with the Pearson *χ*^2^ test or Fisher’s exact test as appropriate. Survival curves were plotted using the Kaplan–Meier method, and the differences were compared using the log-rank test. The hazard ratio (HR) and 95% confidence interval of individual factors associated with disease-free survival and overall survival were evaluated using Cox proportional hazard regression analysis. All data were analyzed using the SPSS 22.0 statistical software package (IBM Corp., USA), and a *p* value < 0.05 was considered statistically significant.

The other materials and methods used in this study are described in Additional file 15.

## Results

### Elevated expression of miR-934 positively correlates with CRLM progression and poor prognosis of patients with CRLM

**Fig. 1:**
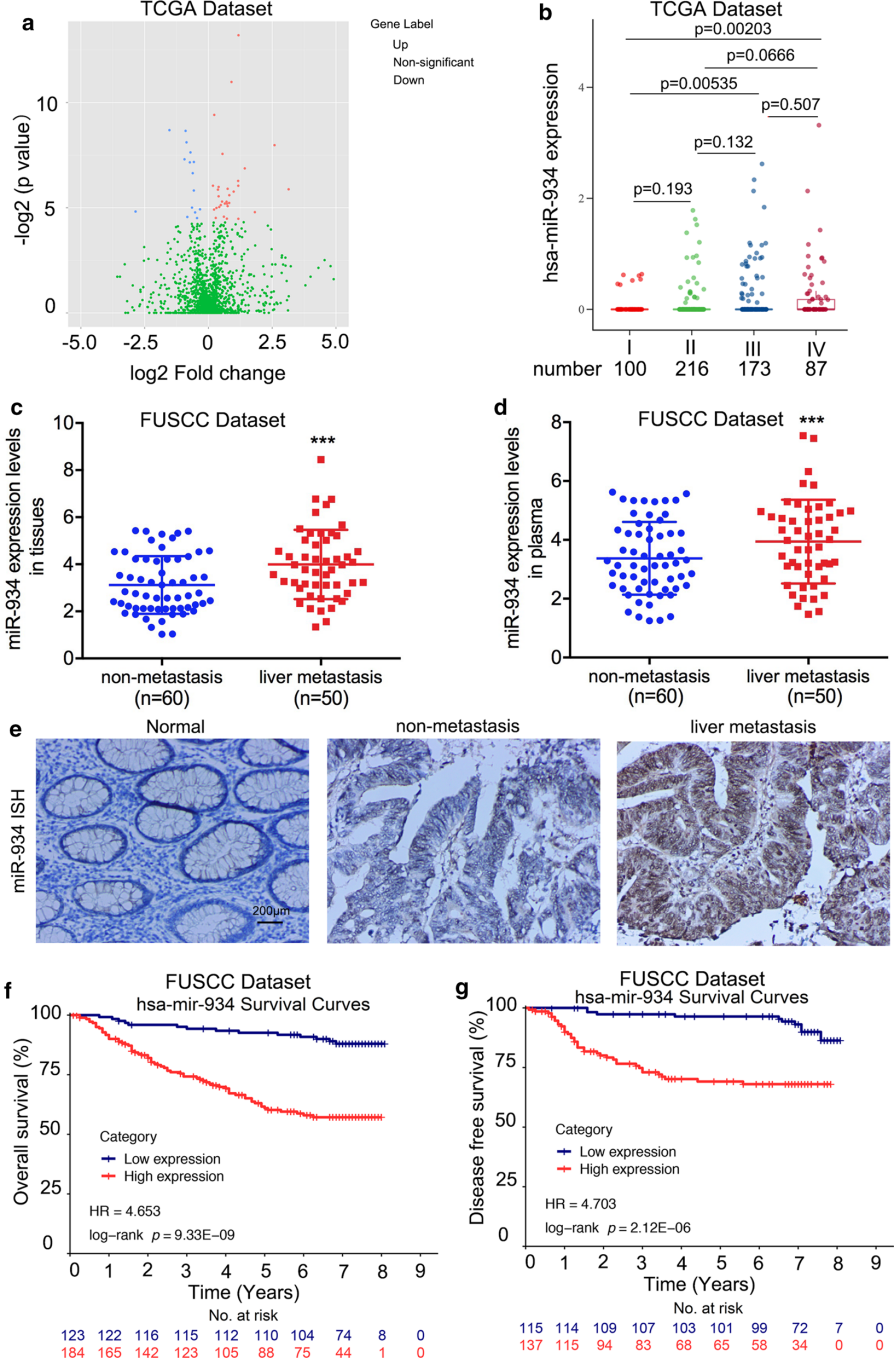
Aberrant expression of miR-934 significantly correlates with liver metastasis and poor prognosis of CRC patients. **a** Volcano plot comparing the miRNA-Seq data between stage I and stage IV CRC patients from the TCGA CC dataset. Each dot represents a microRNA. Dots in red represent microRNAs that are significantly upregulated in stage IV CRC patients (*p* < 0.05 based on DESeq). **b** miR-934 expression in stage I and stage IV CRC patients. Each dot represents a sample. **c**, **d** Expression levels of miR-934 in tissues and serum of two groups of CRC patients classified on the basis of the presence (*n* = 50) or absence (*n* = 60) of liver metastasis. **e** Representative in situ hybridization assay for the detection of miR-934 in normal mucosa (score = 0), CRC tissue without liver metastasis (score = 1), and CRC tissue with liver metastasis (score = 3). **f**, **g** Kaplan–Meier survival analysis with the log-rank test was used to determine the association of miR-934 with OS (**e**) and DFS (**f**) in 308 CRC patients (*p < 0.05; **p < 0.01; ***p < 0.001)

To reveal the potential miRNAs involved in CRLM, we first compared the expression profiles of dysregulated miRNAs between stage I and stage IV CRC tumors using the latest colon adenocarcinoma CRC miRNA-Seq dataset from The Cancer Genome Atlas (TCGA) database. Differential expression analysis based on read counts identified miR-934 as the top miRNA candidate significantly upregulated in stage IV CRCs compared to stage I CRCs (Fig. 1a, b and Additional file 18: Table S3). We further analyzed the expression of the top ten upregulated miRNAs selected from Additional file 18: Table S3 in 20 CRLM and 20 non-CRLM patient’s primary tissues from the FUSCC database and found that miR-934 was also the most significantly upregulated in CRLM compared to non-CRLM (Additional file 1: Fig. S1). To investigate the expression pattern of miR-934 in CRLM, we performed qPCR on 110 pairs of fresh CRC tumor and adjacent normal mucosa tissues. The expression level of miR-934 was found to be significantly higher in CRC tissues than in their corresponding normal mucosa samples (Additional file 2: Fig. S2A). We further investigated miR-934 expression in the serum of 41 healthy controls and 110 CRC patients. We observed that serum from CRC patients exhibited elevated expression of miR-934 compared to that from the control group (Additional file 2: Fig. S2B). Moreover, we divided the 110 CRC tissues into two groups based on the presence or absence of liver metastasis and found that tissue and serum miR-934 expression was upregulated in the liver-metastatic group compared to the non-metastatic group (Fig. 1c, d). Next, to investigate the role of miR-934 in CRLM progression, we compared miR-934 expression in a tissue microarray (TMA) containing 308 CRC samples using ISH and demonstrated that the expression of miR-934 was significantly upregulated in CRC tissues compared with normal mucosa tissues; the increased expression of miR-934 positively correlated with T stage, M stage, advanced AJCC stage, and tumor recurrence, especially in cases of liver metastasis (Fig. 1e and Additional files 19, 20: Tables S4 and S5; *p* < 0.05). The above data indicate that aberrantly high expression of miR-934 is significantly associated with tumor progression and liver metastasis in CRC.

Subsequently, Kaplan–Meier survival analysis with the log-rank test was performed. Both TCGA data and data from our center revealed that patients with higher miR-934 expression had poorer overall survival (OS) and disease-free survival (DFS) rates than those with lower miR-934 expression (both *p* < 0.01, Fig. 1f, g and Additional file 2: Fig. S2C–D). Furthermore, univariate and multivariate survival analyses for OS and DFS also suggested that miR-934 was an independent prognostic biomarker for poor outcome in CRC patients, especially in those with liver metastasis (Additional files 21, 22: Tables S6 and S7). Taken together, our results indicate that high miR-934 expression positively correlates with CRLM progression and poor prognosis in patients with CRLM.

### miR-934 is encapsulated within CRC cell-derived exosomes

**Fig. 2:**
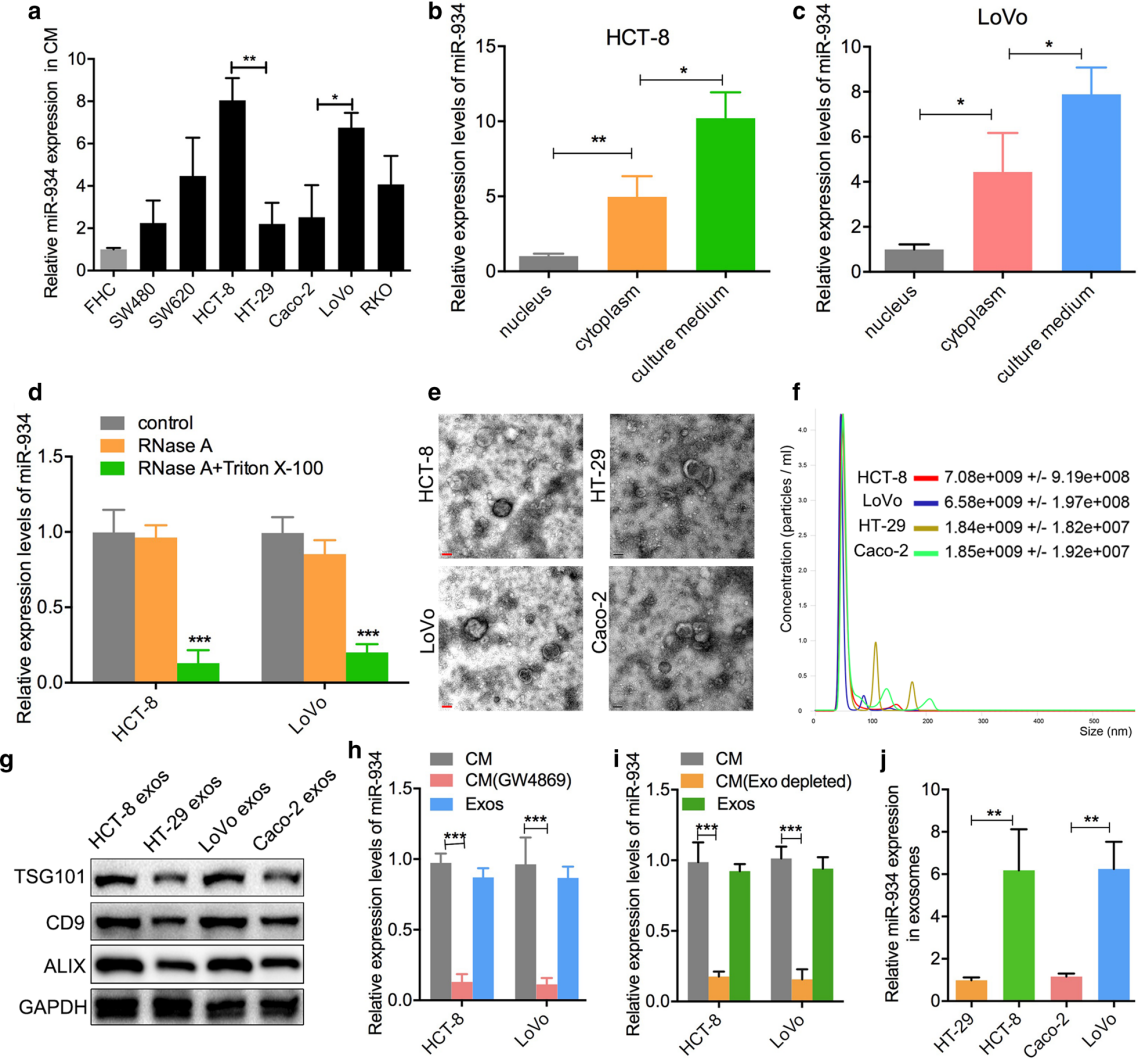
miR-934 is encapsulated within CRC cell-derived exosomes. **a** qPCR analysis of the expression levels of miR-934 in the culture medium (CM) of seven different CRC cell lines and one normal colon cell line. **b**, **c** qPCR analysis of the expression levels of miR-934 in the nucleus, cytoplasm, and culture medium (CM) of the HCT-8 and LoVo cell lines. These cell lines showed relatively higher miR-934 expression than other cell lines. **d** qPCR analysis of the expression levels of miR-934 in the HCT-8 and LoVo cell lines treated with control medium or RNase A (2 mg/mL) alone or in combination with Triton X-100 (0.1%), for 0.5 h. **e**, **f** Phenotype analysis of exosomes derived from HCT-8, HT29, LoVo, and Caco-2 cells using electron microscopy (**e**) and Nano Sight nanoparticle tracking analysis (**f**). **g** Western blot analysis was performed to detect typical exosomal biomarkers (TSG101, CD9, and ALIX) in exosomes derived from the above four CRC cell lines. **h**, **i** qPCR analysis of miR-934 expression in the CM of HCT-8/LoVo cells depleted of exosomes by GW4869 (an inhibitor of exosome secretion) (**h**) or by ultracentrifugation (**i**). **j** qPCR analysis of the expression levels of miR-934 in HT-29/HCT-8/LoVo/Caco-2-derived exosomes (*p < 0.05; **p < 0.01; ***p < 0.001)

To investigate the role of miR-934 in CRLM, we measured miR-934 expression in the culture medium (CM) of seven CRC cell lines and one normal colon cell line (FHC) and found that the expression level of miR-934 in the CM of HCT-8 and LoVo cells was relatively higher than that in the CM of other cell lines (Fig. 2a). Subsequently, we investigated miR-934 expression in the nucleus, cytoplasm, and CM of HCT-8 and LoVo cells. We observed that the CM from HCT-8 and LoVo cells exhibited the highest expression level of miR-934, and the nucleus of these cells displayed the lowest miR-934 expression (Fig. 2b, c). Notably, the level of miR-934 in the CM of CRC cells remained unchanged upon RNase A treatment, while it significantly decreased following treatment with RNase A plus Triton X-100 (Fig. 2d), suggesting that extracellular miR-934 was encased in a membrane and not secreted directly. It has been reported that cancer cells mediate reprogramming of the TME via extracellular vesicle transmission of miRNAs [15]. Therefore, we hypothesized that miR-934 is secreted via exosomes derived from CRC cells. To test this hypothesis, we selected two CRC cell lines with relatively high expression levels of miR-934 (HCT-8 and LoVo) and two CRC cell lines with relatively low expression levels of miR-934 (HT-29 and Caco-2). Exosomes derived from these four CRC cell lines were isolated and purified from the CM. Electron microscopy and nanoparticle tracking analysis demonstrated that more exosomes were secreted by CRC cells that exhibited higher miR-934 expression than by CRC cells that exhibited lower miR-934 expression (Fig. 2e, f). As Hsp70, Tsg101, Aip1, β1-integrin, Cd81, and Cd63 serve as typical biomarkers for exosomes [16], we checked the presence of these proteins in the exosomal extracts using western blotting and found that all the exosomes were positive for the aforementioned biomarkers, which confirmed that the isolated particles were exosomes (Fig. 2g). To elucidate whether miR-934 was mainly derived from the exosomes of CRC cells, we applied GW4869 to inhibit exosome secretion of CRC cells and found that the expression level of miR-934 in CRC cell-derived exosomes was obviously higher than that in exosome-depleted CRC cells (Fig. 2h). In addition, a qPCR assay was performed to analyze miR-934 expression in total CM, exosome-depleted CM (depleted by ultracentrifugation), and exosomes. The results showed that miR-934 expression in total CM or exosomes was significantly higher than that in exosome-depleted CM (Fig. 2i). Furthermore, we investigated miR-934 expression in exosomes derived from the aforementioned four CRC cell lines using qPCR and found that the patterns of miR-934 expression in exosomes were consistent with those in the CM of CRC cells (Fig. 2j), indicating that miR-934 was mainly encapsulated in exosomes derived from CRC cells.

### CRC cell-derived exosomal miR-934 induces M2 polarization of macrophages

**Fig. 3:**
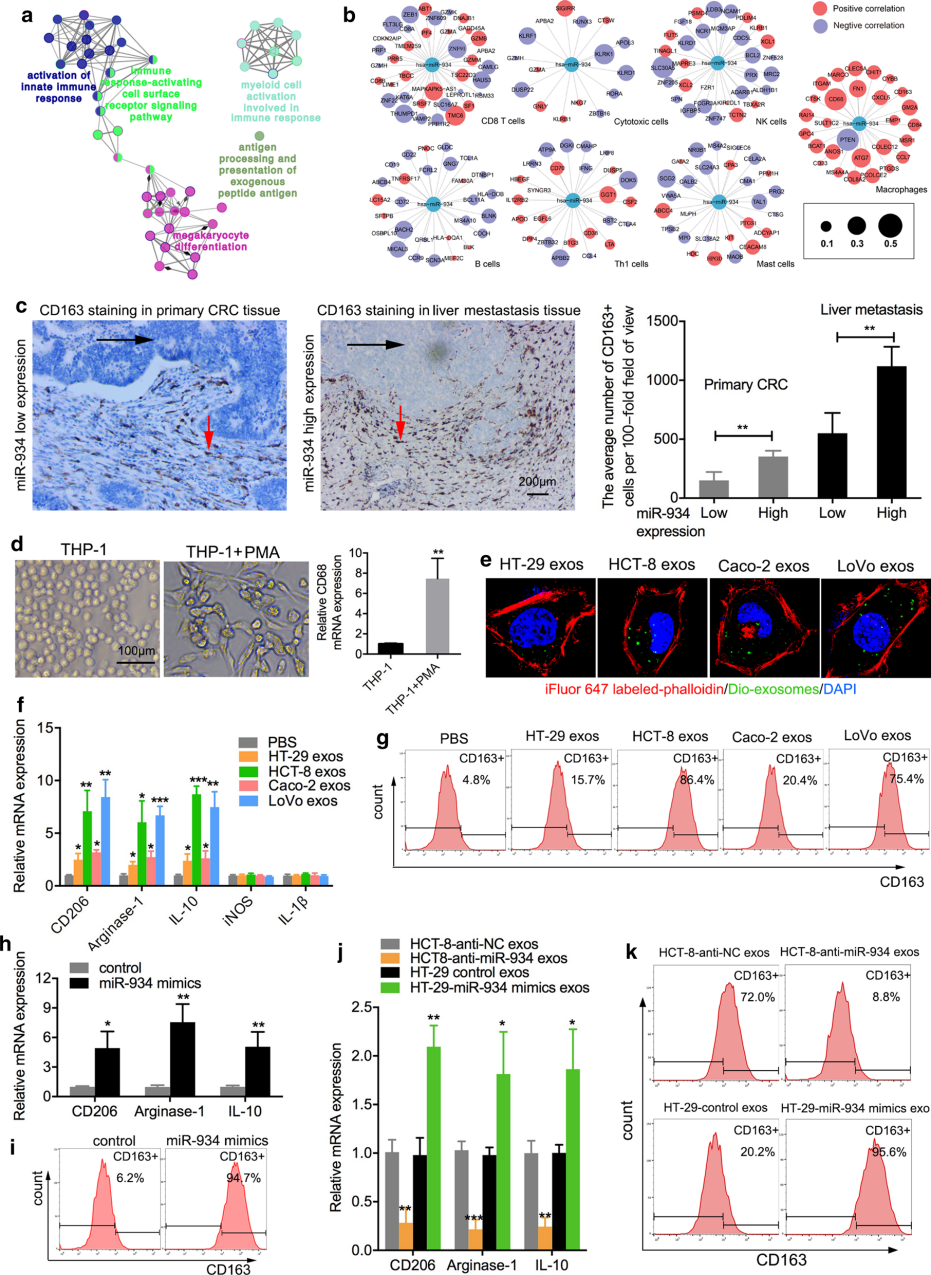
CRC cell-derived exosomal miR-934 induces M2 polarization of macrophages. **a** ClueGO analysis of the 191 genes showing the highest correlation with miR-934 using Cytoscape software. Enriched pathways are shown as nodes interconnected based on the *κ* score. **b** Correlation between miR-934 and specific gene signatures of different immune cells. The node size represents the association *p* value between the neighbor gene and miR-934. **c** IHC staining of TAMs (for the M2 macrophage marker CD163) in primary human CRC tissues and liver-metastatic tissues, n50. The red arrows indicate TAMs; the black arrows indicate tumor cells. Scale bar, 200 μm. The correlation between TAM infiltration and miR-934 expression is also shown. **d** Representative image of macrophages derived from THP-1 cells treated with phorbol 12-myristate 13-acetate (PMA) for 24 h. qPCR analysis of the expression of the macrophage marker CD68 was also performed. **e** Representative immunofluorescence image showing the internalization of DiO-labeled HT-29/HCT-8/Caco-2/LoVo-derived exosomes (green) by PMA-treated THP-1 cells. **f** qPCR analysis of the expression of typical M2 markers (CD206, arginase-1, and IL10) and M1 markers (iNOS and IL-1β) in PMA-pretreated THP-1 cells treated with HT-29/HCT-8/Caco-2/LoVo-derived exosomes or PBS (control) **g** Flow cytometry was performed to analyze the effect of CRC cell-derived exosomes on the expression of the typical M2 marker CD163. qPCR (**h**) and flow cytometry (**i**) were used to determine the effect of exogenous miR-934 on the expression of typical M2 markers in PMA-treated THP-1 cells. qPCR (**j**) and flow cytometry (**k**) were used to determine the effect of exosomes derived from HCT-8 and HT-29 cells transfected with anti-miR-934 on the expression of CD206, arginase-1, IL10, and CD163 (*p < 0.05; **p < 0.01; ***p < 0.001)

To explore the molecular and cellular bases by which miR-934 promotes CRLM, we annotated the cellular functions of 191 miR-934-correlated genes in the TCGA cohort via Cytoscape software and found that miR-934 was extensively involved in multiple inflammatory responses, indicating that miR-934 may be involved in the CRC immune microenvironment (Fig. 3a and Additional file 23: Table S8). Then, by investigating innate immune cells (mast cells, macrophages, and natural killer cells), adaptive immune cells [B cells, T helper 1 (Th1) cells, and CD8 + T cells] and cytotoxic cells, we observed that miR-934 was specifically associated with the gene signatures of macrophages (Fig. 3b). Myeloid-derived macrophages are important innate immune cells that can be induced to differentiate into tumor-associated macrophages (TAMs), i.e., M2-type macrophages, in the TME to mediate the formation of a premetastatic niche [13]. To investigate the correlation between infiltration of TAMs and miR-934 in CRLM, we performed IHC staining to detect the TAM marker CD163 in primary CRC tissues and CRLM tissue samples. As shown in Fig. 3c, we observed elevated expression of miR-934 in areas with abundant CD163^+^ TAM infiltration in CRLM tissues. In addition, Spearman correlation analysis showed that the CD163-positive rate was positively associated with the miR-934 expression level in 50 CRC tissues (Additional file 3: Fig. S3). To further explore whether CRC cell-derived exosomes could induce M2 polarization of macrophages, we employed the human THP-1 cell line as a normal mononuclear macrophage line. THP-1 cells were incubated with PMA to induce differentiation into M0 macrophages, which were appropriately characterized by adherent morphology and elevated expression of the macrophage marker CD68 (Fig. 3d). Next, we examined the effects of CRC cell-derived exosomes on the M2 polarization of macrophages. As shown in Fig. 3e, when cocultured with macrophages, CRC cell-derived exosomes labeled with DiO (green) were internalized by unstained macrophages (Fig. 3e). The expression of the M2 markers [17], (CD206, arginase-1, IL10, and CD163) was significantly increased in macrophages incubated with exosomes derived from CRC cells exhibiting high miR-934 expression compared to that in macrophages incubated with exosomes from cells exhibiting low miR-934 expression or cells incubated with PBS, while M1 markers (iNOS and IL-1β) showed almost no difference (Fig. 3f, g). Consistent with the above results, the expression levels of M2 markers were also significantly increased in human bone marrow-derived macrophages (HBMDMs) treated with exosomes derived from CRC cells with high miR-934 expression compared with those in HBMDMs derived from CRC cells with low miR-934 expression or HBMDMs treated with PBS (Additional file 4: Fig. S4).

To determine whether CRC cell-derived exosomal miR-934 induced M2 polarization of macrophages, we first generated miR-934 mimics and anti-miR-934 constructs to regulate miR-934 expression and confirmed their efficiencies using RT-PCR (Additional file 5: Fig. S5A–B). The expression of M2 markers (CD163, CD206, arginase-1, and IL10) was markedly increased in macrophages transfected with miR-934 mimics compared to control groups (Fig. 3h, i). Furthermore, HCT-8 and HT29 cells were transfected with anti-miR-934 construct, miR-934 mimics, or their negative control vectors, and their exosomes were extracted and added to PMA-treated THP-1 cells. The results showed that the expression of M2 markers (CD206, arginase-1, IL10, and CD163) in PMA-treated THP-1 cells administered exosomes derived from CRC cells transfected with anti-miR-934 or miR-934 mimics were apparently lower or higher, respectively, than that in the control groups (Fig. 3j, k). Taken together, the above results confirm that CRC cell-derived exosomal miR-934 can induce M2 polarization of macrophages.

### hnRNPA2B1 plays a role in the transfer of exosomal miR-934 to macrophages

**Fig. 4:**
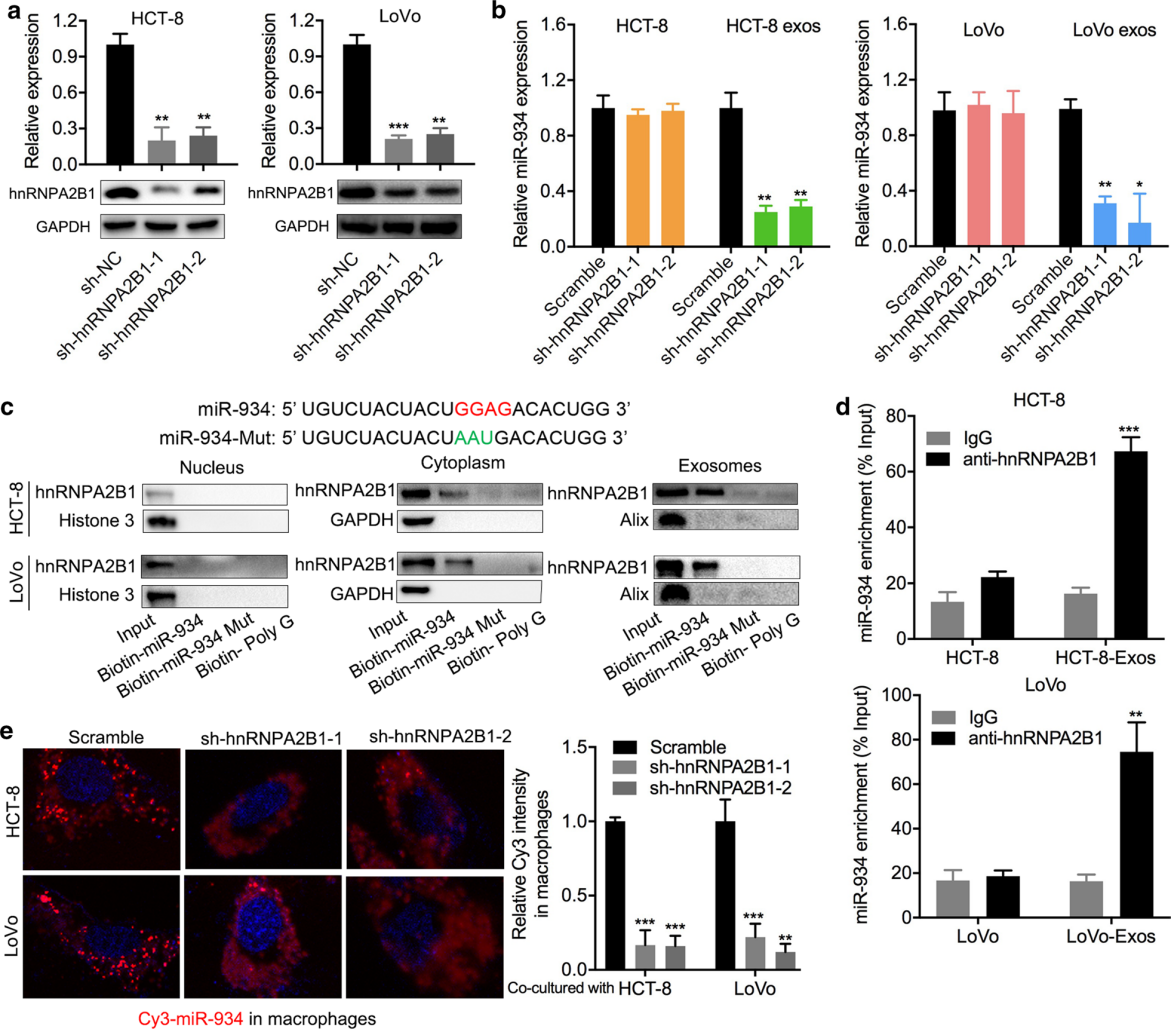
hnRNPA2B1 plays a role in the transfer of CRC cell-derived exosomal miR-934 to macrophages **a** qPCR and western blot analysis of hnRNPA2B1 expression in HCT-8 and LoVo cells after hnRNPA2B1 knockdown. **b** qPCR analysis of miR-934 expression in cell lysates and exosomes derived from hnRNPA2B1-knockdown HCT-8 and LoVo cells. **c** Western blot analysis was performed to determine the association between biotinylated wild-type or mutant miR-934 and hnRNPA2B1 expression in samples derived by miRNA pull-downs performed with nuclear, cytoplasmic, or exosomal extracts from the HCT-8 and LoVo cell lines. Biotinylated poly(G) served as the negative control. **d** RIP assays with anti-hnRNPA2B1 antibody were performed on HCT-8/LoVo-CM and HCT-8/LoVo-derived exosomes. IgG served as the negative control. qPCR was then used to analyze the expression levels of miR-934 in the immunoprecipitated samples, and the expression levels of miR-934 were expressed as percentages with respect to the input sample (% input) in HCT-8 and LoVo cell lines. **e** HCT-8 cells and LoVo cells cotransfected with 100 nM Cy3-labeled miR-934 and sh-hnRNPA2B1 or negative control and then cocultured with PMA-pretreated THP-1 cells for 12 h. An immunofluorescence assay was performed to analyze the effects of hnRNPA2B1 knockdown on the transfer of exosomal (Cy3-labeled) miR-934 from HCT-8 and LoVo cells to macrophages (*p < 0.05; **p < 0.01; ***p < 0.001)

As reported, secretion of exosomal RNAs requires a specific RNA-binding protein for transport [18]. Through RNA pull-down assays and mass spectrometry, we identified three potential RNA-binding proteins for miR-934 transport and found that knockdown of hnRNPA2B1 could decrease the expression of exosomal miR-934 (Fig. 4a, b and Additional file 6: Fig. S6). hnRNPA2B1 is an RNA-binding protein that is involved in transportation and posttranscriptional regulation of miRNAs through binding of the specific motifs GGAG/CCCU [19]. In particular, as there are two GGAG motifs within the miR-934 sequence, we speculated that hnRNPA2B1 may mediate the process of miR-934 packaging into exosomes by binding to the GGAG sequence. Furthermore, miRNA pull-down assays revealed that there was a significant binding relationship between miR-934 and hnRNPA2B1 in the cytoplasm and exosomes; however, this binding could be impaired by mutating the binding sequence (GGAG) of miR-934 (Fig. 4c). RNA immunoprecipitation assays further demonstrated that miR-934 was more enriched in the anti-hnRNPA2B1 antibody group than in the anti-IgG group, both in CRC cells and their exosomal lysates (Fig. 4d). Additionally, knockdown of hnRNPA2B1 inhibited the process of exosomal miR-934 transfer from HCT-8 and LoVo cells to macrophages (Fig. 4e). Therefore, we demonstrated that hnRNPA2B1 could mediate miR-934 packaging into exosomes of CRC cells and transfer of exosomal miR-934 into macrophages by binding to its GGAG sequence.

### Exosomal miR-934 induces M2 polarization of macrophages via downregulation of PTEN expression and activation of the PI3K/AKT signaling pathway

**Fig. 5:**
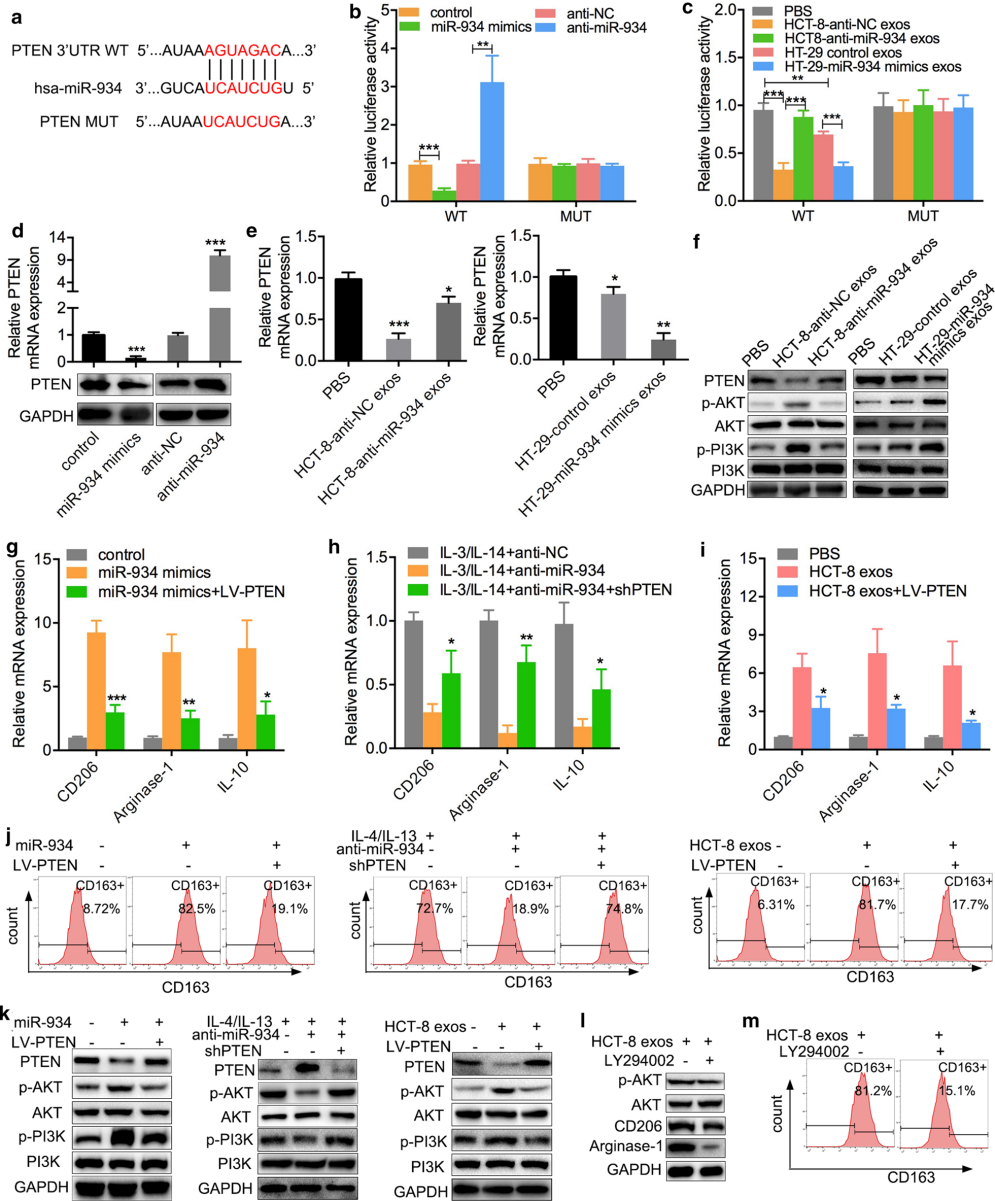
Exosomal miR-934 induces M2 macrophage polarization via downregulation of PTEN expression and activation of the PI3K/AKT signaling pathway. **a** Sequences of the predicted binding sites between miR-934 and the 3′-UTR of the wild-type (WT)/mutant (MUT) PTEN gene. **b** A luciferase reporter gene activity assay was performed to determine the effects of miR-934 mimics or anti-miR-934 on the luciferase activity of the 3′-UTR of the WT/MUT PTEN gene. **c** A luciferase reporter gene activity assay was performed to determine the effect of exosomal miR-934 from HCT-8 and HT-29 cells transfected with miR-934 mimics or anti-miR-934 on the luciferase activity of the 3′-UTR of the WT/MUT PTEN gene in THP-1 cells. **d** The effects of exogenous miR-934 mimics and anti-miR-934 on the expression of PTEN in PMA-treated THP-1 cells were tested using qPCR and western blotting. **e**, **f** The effects of CRC cell-derived exosomal miR-934 on PTEN expression and the expression of PTEN, AKT, p-AKT, PI3k, and p-PI3K in THP-1 cells pretreated with PMA and the aforementioned exosomes were determined using qPCR (**e**) and WB (**f**). **g**, **j** PMA-treated THP-1 cells were transfected with miR-934 mimics, IL3/IL4–anti-miR-934, or HCT-8-derived exosomes and further cocultured with LV-PTEN or shPTEN. The combined effects of exogenous miR-934/LV-PTEN, anti-miR-934/shPTEN, and exosomes/LV-PTEN on the expression of typical M2 markers (CD206, arginase-1, and IL-10) were determined using qPCR (**g**–**i**) and flow cytometry (**j**). **k** Western blot analysis was performed to detect the expression of PI3K/AKT pathway components in PMA-pretreated THP-1 cells transfected with miR-934 mimics, IL3/IL4–anti-miR-934, or HCT-8-derived exosomes. **l**–**m** Western blotting (**l**) and flow cytometry (**m**) were performed to determine the expression of AKT, p-AKT, and M2 markers (CD206, arginase-1, and CD163) in PMA-treated THP-1 cells cocultured with HCT-8 cell-derived exosomes and an inhibitor of PI3K (LY294002) (*p < 0.05; **p < 0.01; ***p < 0.001; scale bar, 200 μm)

We further sought to explore the mechanisms underlying the induction of M2 macrophage polarization by tumor-derived exosomal miR-934. The PTEN/AKT signaling pathway has been widely reported to be involved in macrophage polarization [20]. Data from TargetScan Human 7.2 database demonstrated that there was an alignment between the miR-934 sequence and the 3′-UTR sequence of PTEN, suggesting that miR-934 may target PTEN for the induction of M2 macrophage polarization (Fig. 5a). To verify whether PTEN is a target of miR‐934, CRC cells were cotransfected with miR-934 mimics or anti-miR-934 constructs and their empty vectors; luciferase vectors with the wild-type or mutated version of the binding sites were used for further verification. As shown in Fig. 5b, we transfected miR-934 mimics or anti-miR-934 constructs and their control vectors into PMA-treated THP-1 cells and found that the luciferase activity of the 3′-UTR of PTEN was markedly reduced or increased in the group cotransfected with miR-934 mimics or anti-miR-934 constructs and PTEN wild-type binding site vectors, respectively, while no significant changes were observed in the control or PTEN mutant binding site groups (Fig. 5b). Interestingly, when cells were cocultured with exosomes derived from HCT-8 or HT29 cells transfected with anti-miR-934, miR-934 mimics, or their control vectors, the luciferase activity pattern displayed a trend similar to that observed for PMA-treated THP-1 cells cotransfected with miR-934 mimics or anti-miR-934 and their control vectors (Fig. 5c).

Furthermore, PMA-treated THP-1 cells transfected with miR-934 mimics or anti-miR-934 had downregulated or upregulated the expression of PTEN, respectively, at both the mRNA and protein levels compared to the respective control cells (Fig. 5d). To explore the mechanism by which miR-934 downregulates PTEN expression, exosomes were extracted from HCT-8 or HT29 cells transfected with anti-miR-934 vectors, miR-934 mimics, control vectors, or empty vectors and added to PMA-treated THP-1 cells. We found that PTEN expression in PMA-treated THP-1 cells administered exosomes derived from CRC cells transfected with anti-miR-934 vectors or miR-934 mimics was apparently higher or lower, respectively, than that in the respective control groups (Fig. 5e, f), suggesting that exosome-derived miR-934 can downregulate PTEN expression by targeting its 3′-UTR and activating the PI3K/AKT pathway in PMA-treated THP-1 cells.

To further investigate the effects of PTEN on miR-934-mediated M2 macrophage polarization, plasmids for upregulating or downregulating PTEN expression were transfected into PMA-treated THP-1 cells, and the transfection efficiency was confirmed using western blot (Additional file 7: Fig. S7). As IL-3/IL-4 could induce M2 macrophage polarization [21], we treated THP-1 cells with 50 ng/mL IL-3/IL-4 for 3 days in advance and verified the effect of anti-miR-934 on M2 macrophage polarization. The results demonstrated that overexpression of PTEN partially attenuated the enhancing effect of miR-934 and HCT-8 cell-derived exosomes on the expression of M2 markers (CD206, arginase-1, and IL10), while silencing of PTEN proportionally reversed the attenuation effect of anti-miR-934 on the expression of M2 markers (Fig. 5g, i). Moreover, the expression of the M2 macrophage marker CD163 in PMA-treated THP-1 cells was determined using flow cytometry, and the results were consistent with the aforementioned findings (Fig. 5j). Altogether, our results demonstrate that miR-934 strengthens and anti-miR-934 attenuates the enhancing effect of CRC cell-derived exosomes on the induction of M2 macrophage polarization.

PTEN is a tumor suppressor that regulates multiple cell functions, including cell proliferation and differentiation [22], and PI3K/AKT signaling is one of the most important pathways targeted by PTEN through its phosphatase activity [23]. Therefore, we proposed that the PI3K/AKT signaling pathway could be involved in the induction of M2 macrophage polarization by CRC cell-derived exosomal miR-934. To examine the activation of the PI3K/AKT pathway, we first transfected PMA-treated THP-1 cells with miR-934 mimics or anti-miR-934 or administered HCT-8 cell-derived exosomes. The results of WB showed that overexpression of PTEN could partially attenuate the enhancing effect of miR-934 as well as HCT-8 cell-derived exosomes on the expression of p-AKT and p-PI3K, while silencing of PTEN exhibited the opposite effects (Fig. 5k). More importantly, when cocultured with HCT-8 cell-derived exosomes and an inhibitor of PI3K (LY294002), the expression of p-AKT and M2 markers (CD206, arginase-1, and CD163) in PMA-treated THP-1 cells was downregulated (Fig. 5l, m). Taken together, these results indicate that CRC cell-derived exosomal miR-934 can induce M2 macrophage polarization via downregulation of PTEN expression and activation of the PI3K/AKT signaling pathway.

### M2 macrophage polarization induced by CRC cell-derived exosomal miR-934 promotes CRLM

**Fig. 6:**
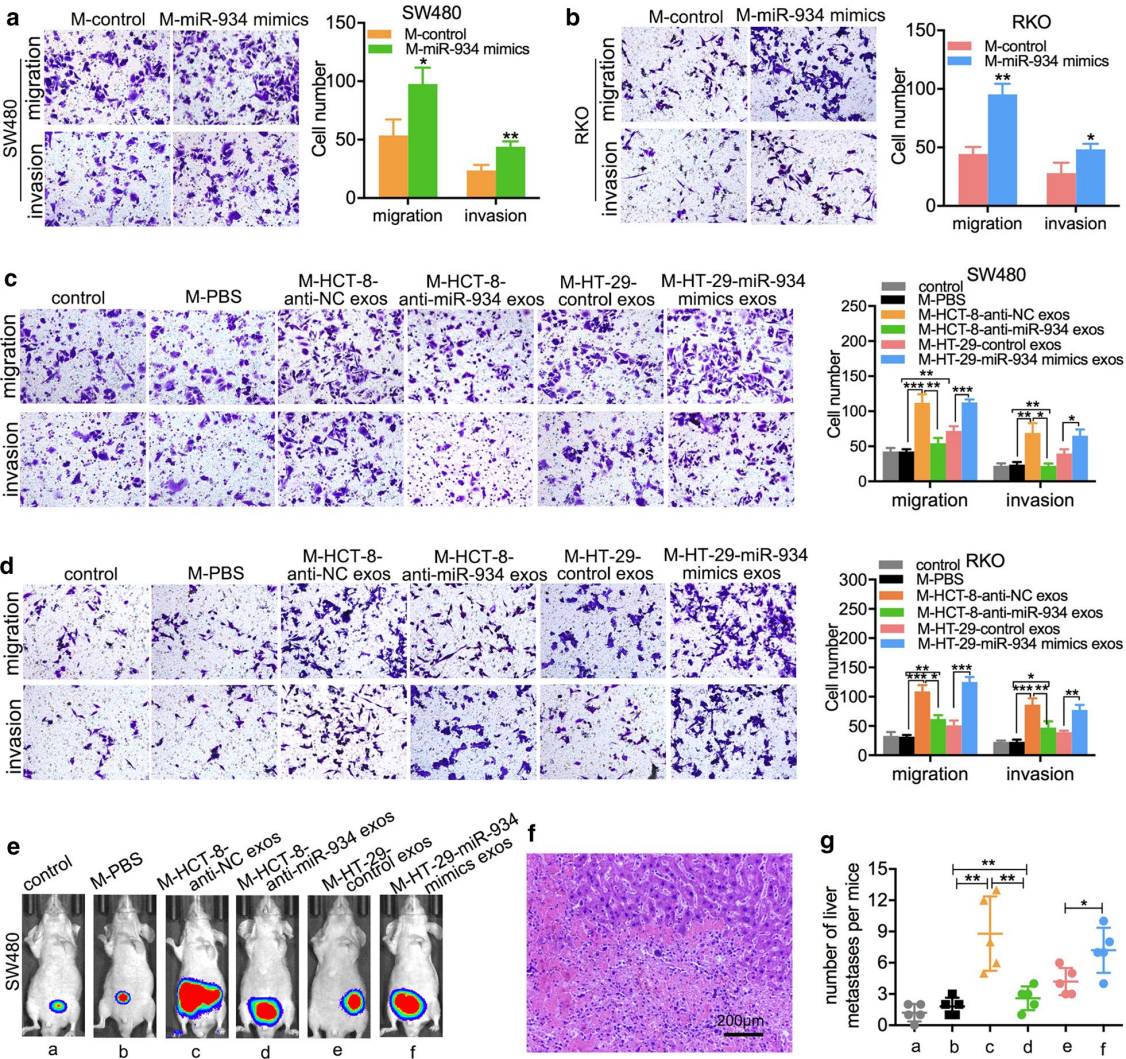
CRC cell-derived exosomal miR-934 induces M2 polarization of macrophages to promote invasion and liver metastasis of CRC cells in vitro and in vivo. **a**, **b** The CRC cell lines SW480 and RKO were cocultured with CM from PMA-treated THP-1 cells for 12 h (THP-1 cells were pretreated with exogenous miR-934 mimics). The effects of exogenous miR-934 on the migratory and invasive abilities of SW480 (**a**) and RKO (**b**) cells were tested using transwell assays. HCT-8 and HT-29 cells were transfected with anti-miR-934 or miR-934 mimics or their controls; exosomes were isolated from all groups. THP-1 cells prestimulated with PMA were treated with these exosomes at a concentration of 25 μg/mL or PBS; then, the CM were obtained and incubated with SW480 or RKO cells for 12 h. Migration and invasion in vitro (**c**, **d**) and liver metastasis in vivo (**e–g**) were evaluated using transwell assays and live imaging combined with HE staining, respectively (*p < 0.05; **p < 0.01; ***p < 0.001)

Numerous studies have reported that the polarization of M2 macrophages induced by tumor-derived exosomes can promote metastasis of CRC [24, 25]. Therefore, we investigated whether the M2 macrophage polarization induced by exosomal miR-934 could also facilitate CRLM. The weakly metastatic CRC cell lines SW480 and RKO were cocultured with CM from THP-1 cells pretreated with exogenous miR-934 mimics. In vitro transwell assays showed that SW480 and RKO cells cocultured with CM from THP-1 cells pretreated with exogenous miR-934 mimics had increased the numbers of migratory and invasive cells compared to the control cells (Fig. 6a, b). Furthermore, we also obtained exosomes from the CRC cell lines HCT-8 and HT-29 transfected with anti-miR-934 or miR-934 mimics and their controls, respectively. Then, we treated THP-1 cells prestimulated with PMA (M0 macrophages) with these exosomes at a concentration of 25 μg/mL and PBS; we subsequently obtained CM from all groups and cocultured SW480 or RKO cells with these CM for 12 h. In vitro and in vivo assays demonstrated that the numbers of migratory and invasive SW480 and RKO cells in groups administered CM from THP-1 cells stimulated with PMA, which were treated with exosomes derived from miR-934-overexpressing or miR-934-downregulated CRC cells, were significantly increased compared with those in the control groups; in addition, the tumor colony growth in the livers of nude mice was decreased by the administration of CM from THP-1 cells stimulated with PMA (Fig. 6c–g and Additional file 8: Fig. S8). These results indicate that the M2 macrophage polarization induced by CRC cell-derived exosomal miR-934 can enhance the invasive and liver-metastatic abilities of CRC cells.

### M2 macrophage polarization induced by CRC cell-derived exosomal miR-934 promotes CRLM via activation of the CXCL13/CXCR5 axis in CRC cells

**Fig. 7:**
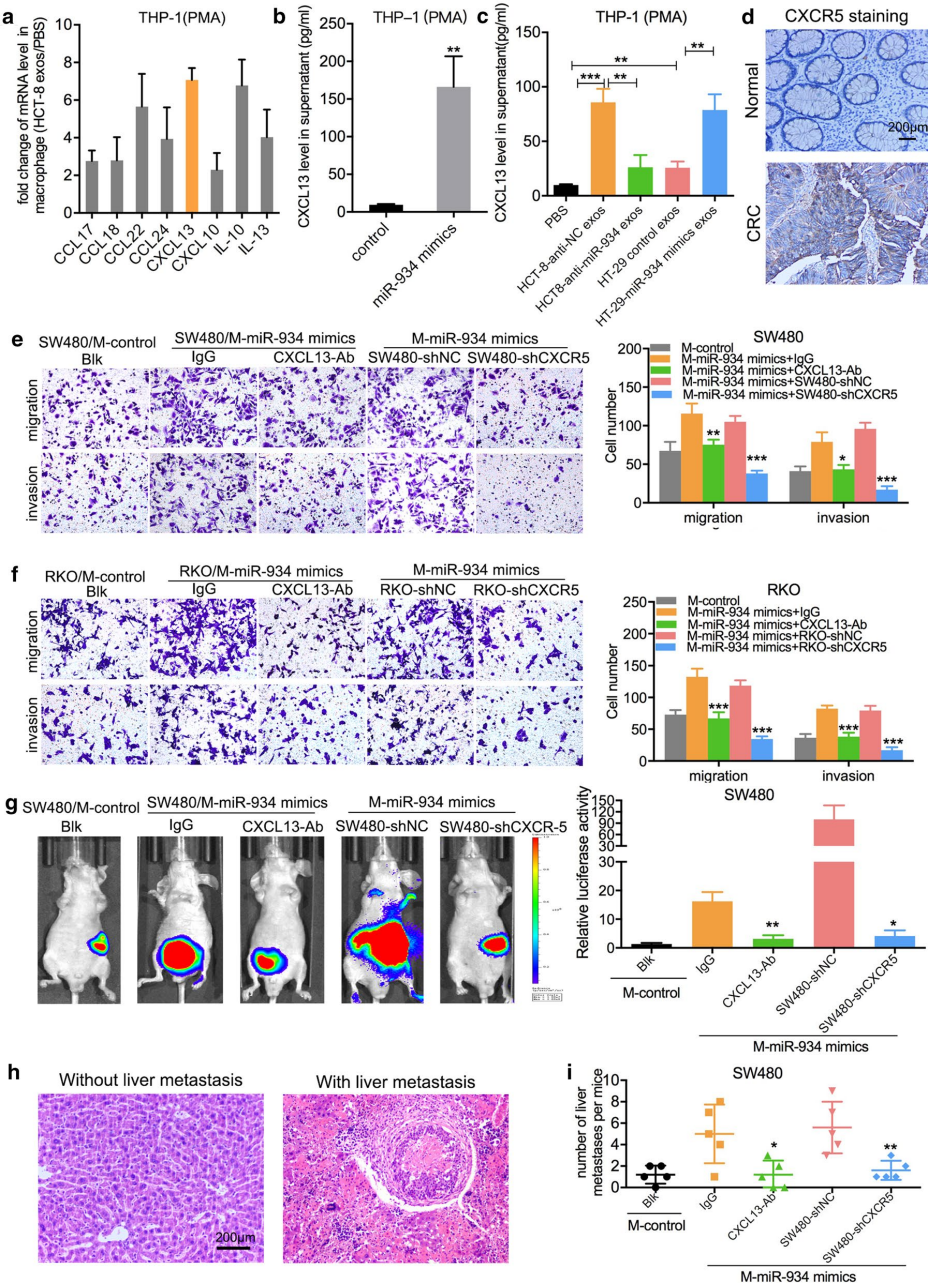
M2 macrophage polarization by exosomal miR-934 promotes invasion and liver metastasis of CRC cells by activating the CXCL13/CXCR5 axis. **a** A human chemokine screening test kit was applied to analyze the levels of chemokines involved in M2 macrophage polarization in PMA-pretreated THP-1 cells treated with HCT-8 cell-derived exosomes. **b** The effect of exogenous miR-934 on the expression of CXCL13 in PMA-treated THP-1 cells was determined using ELISA. **c** ELISAs were used to determine the expression of CXCL13 in PMA-treated THP-1 cells cocultured with exosomes derived from HCT-8 and HT-29 cells, which were transfected with miR-934 mimics or anti-miR-934. **d** Representative IHC staining of CXCR5 in CRC tissues and paired normal tissues. **e**–**i** PMA-treated THP-1 cells were cotransfected with miR-934 mimics and anti-CXCL13 antibody, and then the CM were obtained and cocultured with SW480 or RKO cells for 12 h. SW480/RKO cells were transfected with shCXCR5 and negative control vectors; we obtained CM from PMA-treated THP-1 cells cotransfected with miR-934 mimics and cocultured CXCR5 knockdown or control SW480 or RKO cells with the CM for 12 h. Migration and invasion in vitro (**e**, **f**) and liver metastasis in vivo (**g**–**i**) were evaluated using a transwell assay and live imaging combined with HE staining, respectively (*p < 0.05; **p < 0.01; ***p < 0.001)

The hallmarks of cancer are supported by TAMs, which produce growth factors, extracellular matrix-remodeling molecules, and cytokines to regulate cancer migration and metastasis [26]. Chemokines and cytokines in the tumor immune microenvironment are widely involved in promoting CRC progression, and these can be secreted by M2 macrophages polarized by tumor-derived exosomes. Therefore, we treated PMA-pretreated THP-1 cells with HCT-8 cell-derived exosomes or a negative control and analyzed the levels of chemokines using a human chemokine screening test kit. As shown in Fig. 7a, the CXCL13, IL-10, and CCL22 expression levels were much higher than levels of other chemokines. Then, PMA-pretreated THP-1 cells were transfected with miR-934 mimics or control mimics, and ELISA showed that the expression of CXCL13 was significantly increased (approximately tenfold) compared with that of CCL22 and IL-10 (twofold to threefold), which indicated that CXCL13 might play a significant role in the progression of CRLM (Fig. 7b and Additional file 9: Fig. S9A–B). Furthermore, we extracted exosomes from the CM of HCT8 and HT29 cells transfected with miR-934 mimics, anti-miR-934, or their negative control vectors for ELISA. The results showed that overexpression or knockdown of miR-934 could partially strengthen or attenuate, respectively, the enhancing effects of HT29/HCT8 cell-derived exosomes on the secretion of CXCL13 by M2 macrophages (Fig. 7c). To further understand the effects of exosomal miR-934 on the microenvironment surrounding liver metastases, human Kupffer cells (Life Technologies) were also treated with CRC-derived exosomes, and ELISA showed that exosomal miR-934 could significantly increase CXCL13 secretion by Kupffer cells (Additional file 10: Fig. S10). As CXCL13 is a chemoattractant that promotes chemotaxis of cells expressing CXCR5 [27], we further evaluated the expression level of CXCR5 in CRC tissues and paired normal tissues using IHC staining. We found that CXCR-5 showed stronger staining in CRC tissues than in normal tissues, indicating that CXCL13 secreted by M2-polarized macrophages mainly acted on CRC cells (Fig. 7d). To investigate the role of the CXCL13/CXCR5 axis in CRLM, we generated a short hairpin (sh)CXCR5 construct to regulate CXCR5 expression in CRC cells and confirmed its efficiency using WB (Additional file 11: Fig. S11). PMA-pretreated THP-1 cells were transfected with miR-934 mimics and cocultured with SW480 cells or RKO cells with anti-CXCL13 antibody or IgG added into the CM. Additionally, the above THP-1 cells were cocultured with SW480 cells or RKO cells after knocking down CXCR5. In vitro transwell assays revealed that the anti-CXCL13 antibody inhibited CRC cell migration and invasion in groups that were cocultured with CM from miR-934-overexpressing PMA-treated THP-1 cells; the migratory and invasive abilities were reduced in SW480 and RKO cells transfected with shCXCR5 when they were cocultured with miR-934-overexpressing PMA-treated THP-1 cells (Fig. 7e, f). Furthermore, in vivo liver metastasis assays demonstrated that CRC cells treated with anti-CXCL13 antibody or with knockdown of CXCR5 had reduced liver metastatic colonies compared to the control groups (Fig. 7g–i and Additional file 12: Fig. S12). To further confirm the effects of CXCL13, we isolated CD163+ or CD206+ macrophages (TAMs) from primary CRC tissues and then cocultured them with SW480 or RKO cells with the anti-CXCL13 antibody added to CM or cocultured them with SW480 or RKO cells transfected with shCXCR5. Transwell assays showed that both the anti-CXCL13 antibody and knockdown of CXCR5 in CRC cells reduced the migration and invasion induced by TAMs in SW480 cells and RKO cells (Additional file 13: Fig. S13). All the above data indicate that the M2 macrophage polarization induced by exosomal miR-934 can promote CRLM by inducing secretion of CXCL13 and activating the CXCL13/CXCR5 axis in CRC cells.

To further clarify the effects of miR-934, CXCL13, and CXCR5 on CRLM, we performed qPCR and IHC assays in 50 cases of primary CRC tissues and paired liver metastatic tissues. The results showed that the staining of miR-934, CXCL13, and CXCR5 was stronger in the miR-934 high group than in the miR-934 low group in both primary CRC tissues and paired liver metastatic tissues. In addition, the expression of CXCL13 and CXCR5 was higher in metastasis than adjacent liver tissues (Additional file 14: Fig. S14). The correlation analysis showed that miR-934 expression was positively associated with CXCL13 and CXCR5 expression both in primary CRC tissues and in paired liver metastatic tissues (Additional file 24: Tables S9 and S10). These results highlight that miR-934 expression-mediated CXCL13/CXCR5 axis activation plays significant roles in CRLM.

### CXCL13/CXCR5/NFκB/p65/miR-934 positive feedback loop mediates the interaction between M2 macrophages and CRC cells during CRLM

**Fig. 8:**
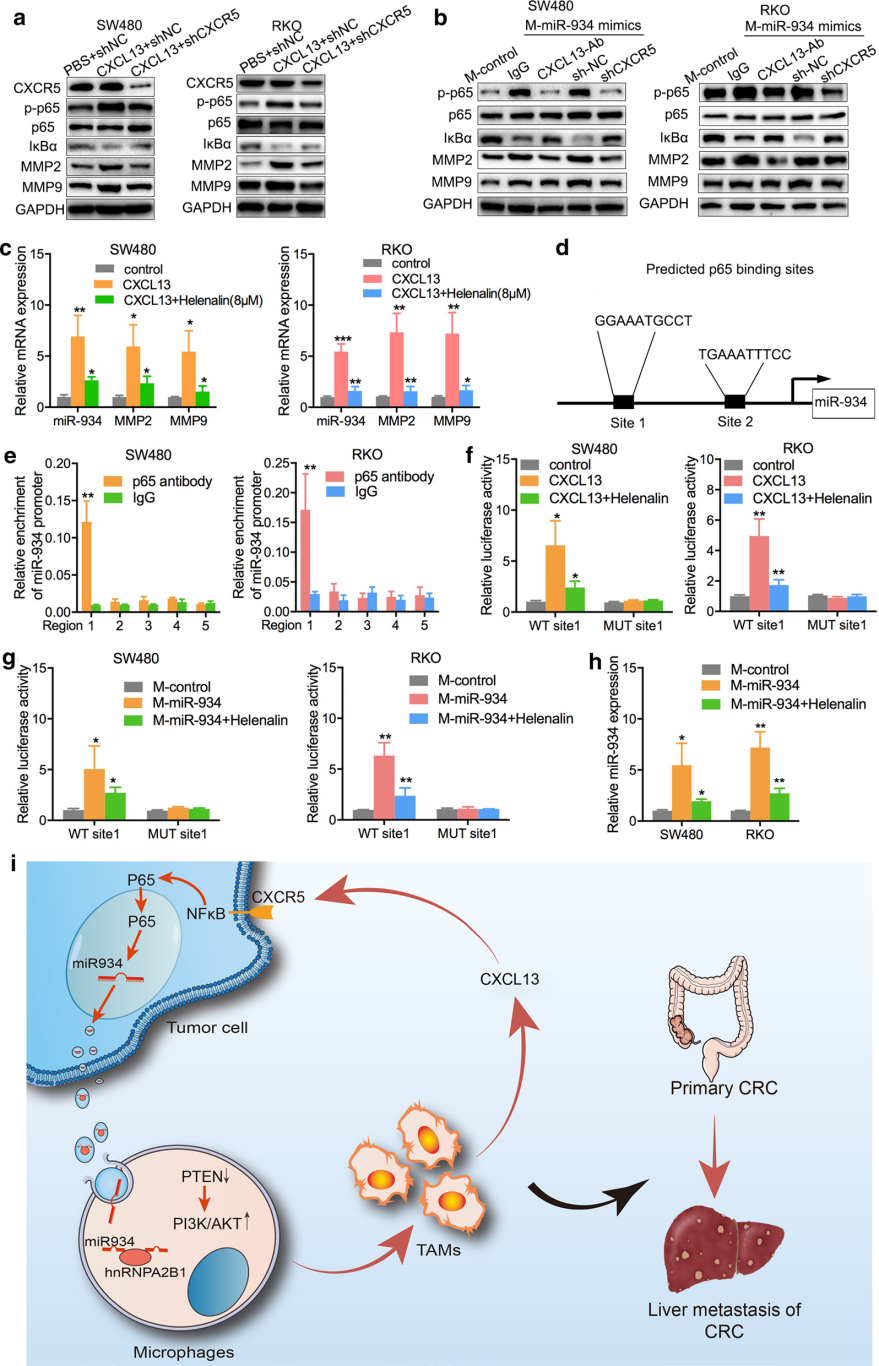
CXCL13/CXCR5/NFκB/p65/miR-934 positive feedback loop mediates the interaction between M2 macrophages and CRC cells during CRLM. **a** Western blot assays were performed to evaluate the expression levels of total p65, phosphorylated p65, IκBα, MMP2, and MMP9 in SW480 and RKO cells pretransfected with exogenous CXCL13 or shCXCR5. **b** PMA-treated THP-1 cells were transfected with miR-934 mimics and treated with anti-CXCL13 antibody or transfected with shCXCR5. The CM of these THP-1 cells was added to SW480 and RKO cells, and the expression of p65, phosphorylated p65, IκBα, MMP2, and MMP9 was determined again using western blot assay. **c** SW480 and RKO cells were transfected with exogenous CXCL13 and cocultured with or without an inhibitor of NFκB/p65 signaling (helenalin), and the expression of miR-934, MMP2, and MMP9 was examined using qPCR. **d** Schematic diagram representing the two specific binding sites between p65 and the promoter of miR-934. **E.** A ChIP assay was used to evaluate five potential binding regions between p65 and the miR-934 promoter in SW480 and RKO cells via LASAGNA-Search 2.0 and the human genomic databases of the National Center for Biotechnology Information (NCBI). **f** Mutants with truncated binding sites and their control vectors were cloned into pGL3-luciferase reporter plasmids and then transfected into SW480 and RKO cells. **g** A luciferase reporter gene activity assay was used to analyze the effects of helenalin (a p65 inhibitor) as well as the CM of miR-934-overexpressing THP-1 cells (PMA pretreatment) on the luciferase activity of the WT or MUT p65 binding site 1 of miR-934 promoter constructs in SW480 and RKO cells. **h** qPCR assays were performed to analyze the effects of helenalin on the RNA expression levels of miR-934 in SW480 and RKO cells cocultured with the culture medium of PMA-treated THP-1 cells treated with miR-934. **i** Graphical illustration of the interaction between TAMs and CRC cells (*p < 0.05; **p < 0.01; ***p < 0.001)

It has been widely reported that the CXCL13/CXCR5 axis activates the canonical NFκB pathway and amplifies inflammatory responses in several cancer cells [28, 29]. We hypothesized that CXCL13/CXCR5 was involved in regulating the NFκB signaling pathway in CRC cells. As shown in Fig. 8a, WB revealed that CXCL13 promoted p65 phosphorylation, and the expression of NFκB, MMP2, and MMP9 was upregulated while the expression of IκBα was downregulated in SW480 and RKO cells compared to the control groups. Downregulation of CXCR5 expression could partially attenuate the enhancing effect of CXCL13. Furthermore, PMA-treated THP-1 cells were pretreated with miR-934 mimics and then cocultured with SW480 cells or RKO cells with anti-CXCL13 antibody added into the CM or cocultured with SW480 cells or RKO cells transfected with shCXCR5. We found that the anti-CXCL13 antibody or knockdown of CXCR5 in CRC cells could inhibit the activation of the NFκB signaling pathway induced by the CM of miR-934-overexpressing PMA-treated THP-1 cells (Fig. 8b). Notably, following inhibition of NFκB/p65 signaling by helenalin, the expression of miR-934, MMP2, and MMP9 was significantly lower in SW480 and RKO cells than in the control groups (Fig. 8c), which suggested that miR-934 may also be positively regulated by NFκB/p65 signaling through a positive feedback loop.

As p65 translocates into the nucleus and promotes transcription of its targeted genes, it was hypothesized that activation of the NFκB/p65 signaling pathway could upregulate miR-934 expression through the transcriptional activities of p65. To test this hypothesis, we first predicted the potential transcriptional binding regions of and specific binding sites between p65 and miR-934 via LASAGNA-Search 2.0 and the human genomic databases of the National Center for Biotechnology Information (NCBI). We identified five potential transcriptional binding regions and two specific binding sites between p65 and the promoter of miR-934 (Fig. 8d). Subsequently, we transfected SW480 and RKO cells with various vectors that contained p65-binding regions in the promoter of miR-934; ChIP assay demonstrated that transcription of miR-934 by p65 was significantly higher within the region between − 2002 and − 1500 bp (region 1), and no obvious changes were observed in the other four binding regions (Fig. 8e). These data indicate that region 1 might play a key role in regulating the induction of miR-934 transcription mediated by p65 in CRC cells. Taking into consideration the two binding sites between p65 and the miR-934 promoter in region 1, we further constructed wild-type and mutant binding sites and performed a luciferase reporter gene assay to confirm the transcriptional effect of p65 on the promoter of miR-934 in SW480 and RKO cells (Fig. 8f and Additional file 25: Table S11). We discovered that only mutant binding site 1 could downregulate the luciferase reporter gene activity of p65 and inhibit the transcriptional expression of miR-934, which demonstrated that p65 could positively and directly regulate miR-934 expression through binding site 1 (Fig. 8f). Moreover, we demonstrated that mutant binding site 1 in SW480 and RKO cells could abolish the promoting role of CXCL13 and the inhibiting role of NFκB/p65 signaling on miR-934 expression (Fig. 8g, h). Taken together, these results demonstrate that the CXCL13/CXCR5/NFκB/p65 signaling pathway promotes the transcription of miR-934 in CRC cells, forming a positive feedback loop that is involved in persistent M2 macrophage polarization and in the invasion and metastasis of CRC cells.

## Discussion

CRLM is one of the most common secondary liver cancers and is mediated by interactions between tumor cells and the TME in the liver. The tumor premetastatic niche, which is a dynamic system regulated by intercellular interactions, is responsible for tumor progression and distant metastasis. In the present study, we demonstrated that miR-934 was highly expressed in CRLM and significantly correlated with the poor prognosis of CRLM patients. Importantly, we revealed that miR-934-enriched exosomes could promote CRLM by mediating the crosstalk between colorectal cancer cells and TAMs and forming a metastatic microenvironment. The exosomal miR-934-regulated interaction between tumor cells and TAMs reveals the molecular mechanism of CRLM and explains why all CRC patients do not develop liver metastasis.

Accumulating evidence has revealed that tumor-derived exosomes frequently transfer miRNAs to recipient cells to induce repression of the target genes [10, 24]. However, fewer studies have focused on the transfer of CRC cell-derived exosomal miRNAs to macrophages in the liver and their subsequent role in the malignancy of recipient cells during CRLM. Although miR-934 has been reported to be aberrantly overexpressed in human bladder cancer, ovarian cancer, and alcohol-associated head and neck squamous cell carcinoma, contributing to the development and progression of these cancers [30–32], the specific functions of exosomal miR-934 and the molecular mechanisms underlying the promotion of CRLM by miR-934 are yet to be revealed. Our results explain why different types of cancer show different metastatic capabilities. Notably, serum samples from CRC patients, particularly CRLM patients, exhibited apparently higher levels of exosomal miR-934 than serum samples from normal controls, which might aid in the development of liquid biopsy techniques in the future.

Owing to the wide landscape of genomic alterations and limited therapeutic success in targeting tumor cells, recent studies have focused on better understanding and possibly targeting the microenvironment in which tumors develop [33]. TAMs are among the most common tumor stromal cells in the TME and play significant roles in modulating the growth and metastasis of tumors. Exosomes have also been identified in the TME, and increasing evidence suggests that exosomes carry elements that could modulate metastasis, angiogenesis, and drug resistance [15]. In the TME, exosomes horizontally transfer functional biomolecules, including miRNAs, to recipient cells. TAMs are the most abundant cells in the TME [25, 34], and multiple studies have proved that exosomal miRNAs promote tumorigenesis and tumor progression by regulating the interaction between tumor cells and TAMs. [24, 35] For example, exosome-encapsulated miRNAs contribute to CXCL12/CXCR4-induced CRLM by enhancing M2 polarization of macrophages [24], and hypoxic epithelial ovarian cancer-derived exosomal miR-940 promotes epithelial ovarian cancer proliferation and migration by inducing M2 polarization of macrophages [35]. In our study, we demonstrated that hnRNPA2B1 could mediate the packaging of miR-934 into exosomes of CRC cells and the transfer of CRC cell-derived exosomal miR-934 to macrophages by binding to the GGAG sequence of miR-934. Furthermore, CRC cell-derived exosomal miR-934 induced M2 macrophage polarization via downregulation of PTEN expression and activation of the PI3K/AKT signaling pathway. This is consistent with the report that exosomal miRNAs could be efficiently delivered into target cells to regulate their biology [36].

M2 polarization of TAMs plays an essential role in regulating tumor growth, migration, and angiogenesis by leading to the production of growth factors and cytokines by macrophages [26]. Chemokines, which belong to the cytokines family, act as chemoattractants to induce the migration of responding cells [29]. The chemokine ligand CXCL13 and its chemokine receptor CXCR5 are among the key chemotactic factors that play crucial roles in the biology of cancer cells, and the CXCL13/CXCR5 signaling axis makes pivotal contributions to the development and progression of several human cancers [37–39]. The CXCL13/CXCR5 axis promotes tumor progression through the PI3K/AKT/mTOR pathway, contributes to poor prognosis in clear cell renal cell carcinoma [38], and promotes the growth and invasion of colon cancer cells via the PI3K/AKT pathway [39]. In the present study, we demonstrated that the M2 polarization of macrophages induced by CRC cell-derived exosomal miR-934 could promote CRLM via a CXCL13/CXCR5/NFκB/p65 positive feedback loop, which resulted in persistent crosstalk between tumor cells and TAMs, forming an inflammatory microenvironment to foster CRLM. Our study highlighted that the development of CRLM is a complex process involving multiple molecular interactions regulated by key genes.

## Conclusions

We demonstrated that CRC-derived exosomal miR-934 could induce M2 macrophage polarization via downregulation of PTEN expression and activation of the PI3K/AKT signaling pathway. In addition, we revealed that exosomal miR-934-polarized M2 macrophages could promote CRLM via a CXCL13/CXCR5/NFκB/p65/miR-934 positive feedback loop. Thus, our study elucidated a novel molecular mechanism promoting CRLM underlying the interaction between tumor cells and TAMs, which will contribute to the development of effective preventive and therapeutic strategies for CRLM. More importantly, high miR-934 expression in serum exosomes was correlated with CRLM, suggesting it may be a promising biomarker for liquid biopsy and predicting the risk of CRLM in the future. In addition, targeting exosomal miR-934-mediated crosstalk between tumor cells and TAMs may provide novel strategies for the treatment of CRLM.

## Supplementary information

**Additional file 1: Figure S1**. miR-934 is the top miRNA upregulated in CRLM compared to non-CRLM samples’ primary tumor tissues. qPCR analysis of the expression of the top ten upregulated miRNAs in 20 CRLM and 20 non-CRLM samples’ primary tumor tissues from the FUSCC database.

**Additional file 2: Figure S2**. Levels of miR-934 in the tissues and serum of CRC patients from the FUSCC dataset and the role of miR-934 in predicting the OS and DFS of CRC patients from the TCGA dataset. **a** Expression of miR-934 in 41 normal tissues and 110 CRC tissues. **b** Expression of miR-934 in the serum of 41 healthy controls and 110 CRC patients. **c, d**. Kaplan–Meier survival analysis with the log-rank test was used to determine the association of miR-934 expression with the OS (**c**) and DFS (**d**) of CRC patients from the TCGA dataset (*p < 0.05; **p < 0.01; ***p < 0.001).

**Additional file 3: Figure S3**. Spearman correlation analysis of the CD163 positivity rate and miR-934 expression in 50 CRC tissues. Spearman correlation analysis showed that the CD163 positivity rate was positively associated with miR-934 expression in 50 CRC tissues.

**Additional file 4: Figure S4**. Effects of exosomal miR-934 on the polarization of HBMDMs. **a** Morphology of human bone marrow-derived macrophages (HBMDMs). The macrophage marker CD11b was measured with flow cytometry. **b** qPCR analysis of the changes in M2 marker (CD163, CD206, Arginase-1, and IL-10) expression levels after HBMDMs were treated with CRC cell-derived exosomes (*p < 0.05; **p < 0.01; ***p < 0.001).

**Additional file 5: Figure S5**. Effect of miR-934 mimics and anti-miR-934 vectors on THP-1 cells prestimulated with PMA and CRC cells. **a** Levels of miR-934 in THP-1 cells prestimulated with PMA and transfected with miR-934 mimics, anti-miR-934 vectors, or their control vectors. **b** Levels of miR-934 in HT-29 and HCT8 cells transfected with miR-934 mimics, anti-miR-934 vectors, or their control vectors (**p < 0.01; ***p < 0.001).

**Additional file 6: Figure S6**. Effect of the RNA binding proteins hnRNPU and hnRNPR on the expression of CRC cell-derived exosomal miR-934. **a, b** Changes in the mRNA and protein expression levels of hnRNPU and hnRNPR induced by transfection of their knockdown plasmids. **c, d** Levels of total and exosomal miR-934 after transfection of shhnRNPU, shhnRNPR, or their negative control plasmids into HCT8 and LoVo cells (*p < 0.05; **p < 0.01; ***p < 0.001).

**Additional file 7: Figure S7**. Changes in the expression of PTEN induced by transfection of overexpression (**a**) or knockdown (**b**) vectors into PMA-treated THP-1 cells.

**Additional file 8: Figure S8**. Representative HE staining images of each group (as a supplement to Fig. 6e).

**Additional file 9: Figure S9**. Changes in the secretion of CCL22 and IL-10 after THP-1 cells prestimulated with PMA were transfected with miR-934 mimics. ELISA assays examined CCL22 (**a**) and IL-10 (**b**) in the CM of THP-1 cells prestimulated with PMA and transfected with miR-934 mimics (*p < 0.05).

**Additional file 10: Figure S10**. Effects of exosomal miR-934 on the secretion of CXCL13 by Kupffer cells. ELISA measuring CXCL13 in the CM of human Kupffer cells pretreated with exosomes derived from HCT-8/HT-29 cells and transfected with anti-miR-934 or miR-934 mimics (*p < 0.05; **p < 0.01).

**Additional file 11: Figure S11**. Changes in the expression of CXCR5 induced by transfection of its knockdown vectors into SW480 cells.

**Additional file 12: Figure S12**. Representative HE staining images of each group (as a supplement to Fig. 7g).

**Additional file 13: Figure S13**. TAMs promote the migration and invasion of CRC cells by activating the CXCL13/CXCR5 axis. a, b TAMs were cocultured with CRC cells and anti-CXCL13 antibody or cocultured with CRC cells transfected with sh-CXCR5. Migration and invasion in vitro were evaluated using a transwell assay (*p < 0.05; **p < 0.01; ***p < 0.001).

**Additional file 14: Figure S14**. Association of miR-934 expression in CRC tissues and paired liver metastatic tissues with CXCL13 and CXCR5 expression in CRC tissues, adjacent normal liver tissues and paired liver metastatic tissues. **a** A qPCR assay was used to examine miR-934 expression in 50 CRC tissues and paired liver metastatic tissues. **b** Representative images of IHC staining of CXCL13 and CXCR5 in 50 CRC tissues, adjacent normal liver tissues and paired liver metastatic tissues (*p < 0.05; The red arrows indicate paired liver metastatic tissues; the black arrows indicate adjacent normal liver tissues; Scale bar, 200 μm).

**Additional file 15:** Supplementary materials and methods.

**Additional file 16: Table S1**. Data of sequences for qPCR and cell transfection in this study.

**Additional file 17: Table S2**. Data of antibodies used in our research

**Additional file 18: Table S3**. Deregulated miRNA between stage I and stage IV colon cancer from TCGA data set.

**Additional file 19: Table S4**. Expression of miR-934 in normal colorectal mucosa and primary cancerous tissues (*n* = 308)

**Additional file 20: Table S5**. Associations between miR-934 expression and clinicopathological characteristics in 308 CRC patients.

**Additional file 21: Table S6**. Univariate and multivariate analysis of overall survival in 308 CRC patients

**Additional file 22: Table S7**. Univariate and multivariate analysis of disease-free survival in 308 CRC patients.

**Additional file 23: Table S8**. The top 191 miR-934-correlated genes in TCGA cohorts.

**Additional file 24: Table S9**. Association of miR-934 expression with CXCL13 and CXCR5 expression in CRC tissues (n = 50). **Table S10:** Association of miR-934 expression with CXCL13 and CXCR5 expression in paired liver metastatic tissues (*n* = 50).

**Additional file 25: Table S11**. The wild (WT) and mutant (MUT) sequences of binding sites between p65 and the promoter of miR-934.

## Data Availability

The datasets used and analyzed during the current study are available within the manuscript and its additional files.

## Abbreviations

miRNAs: MicroRNAs
TAMs: Tumor-associated macrophages
TME: Tumor microenvironment
CRC: Colorectal cancer
CRLM: Liver metastasis of CRC
UTR: Untranslated region
AJCC: American Joint Committee on Cancer
TCGA: The Cancer Genome Atlas
TMA: Tissue microarray
CM: Culture medium
HBMDMs: Human bone marrow-derived macrophages
