# Correction to: Tumor-derived exosomal miR-934 induces macrophage M2 polarization to promote liver metastasis of colorectal cancer

**DOI:** 10.1186/s13045-021-01042-0

**Published:** 2021-02-23

**Authors:** Senlin Zhao, Yushuai Mi, Bingjie Guan, Binbin Zheng, Ping Wei, Yanzi Gu, Zhengxiang Zhang, Sanjun Cai, Ye Xu, Xinxiang Li, Xuefeng He, Xinyang Zhong, Guichao Li, Zhiyu Chen, Dawei Li

**Affiliations:** 1 Department of Colorectal Surgery, Fudan University Shanghai Cancer Center, 270 Dong’an Road, Shanghai, 200032 China; 2 Department of Oncology, Shanghai Medical College, Fudan University, 270 Dong’an Road, Shanghai, 200032 China; 3 Department of Gastrointestinal Surgery, The Second Hospital, Cheeloo College of Medicine, Shandong University, No. 247 Beiyuan Street, Jinan, 250033 China; 4 Department of General Surgery, Shanghai General Hospital, School of Medicine, Shanghai Jiaotong University, 85 Wujin Road, Shanghai, 200080 China; 5 Cancer Institute, Fudan University Shanghai Cancer Center, 270 Dong’an Road, Shanghai, 200032 China; 6 Department of Pathology, Fudan University Shanghai Cancer Center, 270 Dong’an Road, Shanghai, 200032 China; 7 Department of Biobank, Fudan University Shanghai Cancer Center, 270 Dong’an Road, Shanghai, 200032 China; 8 Department of Oncology, Yijishan Hospital of Wannan Medical College, No. 2 Zheshan Road, Wuhu, 241001 Anhui China; 9 Department of Radiation Oncology, Fudan University Shanghai Cancer Center, 270 Dong’an Road, Shanghai, 200032 China; 10 Department of Medical Oncology, Fudan University Shanghai Cancer Center, 270 Dong’an Road, Shanghai, 200032 China

## Correction to: J Hematol Oncol 10.1186/s13045-020-00991-2

It has come to the authors’ attention that an incorrect image had been inadvertently included in the paper. The correct version of Fig. 2g is shown corrected as ahead. This correction has not changed the description, interpretation, or the original conclusions of the article. The authors apologize for these errors and any consequent inconvenience to authors and readers.

**Fig. 2:**
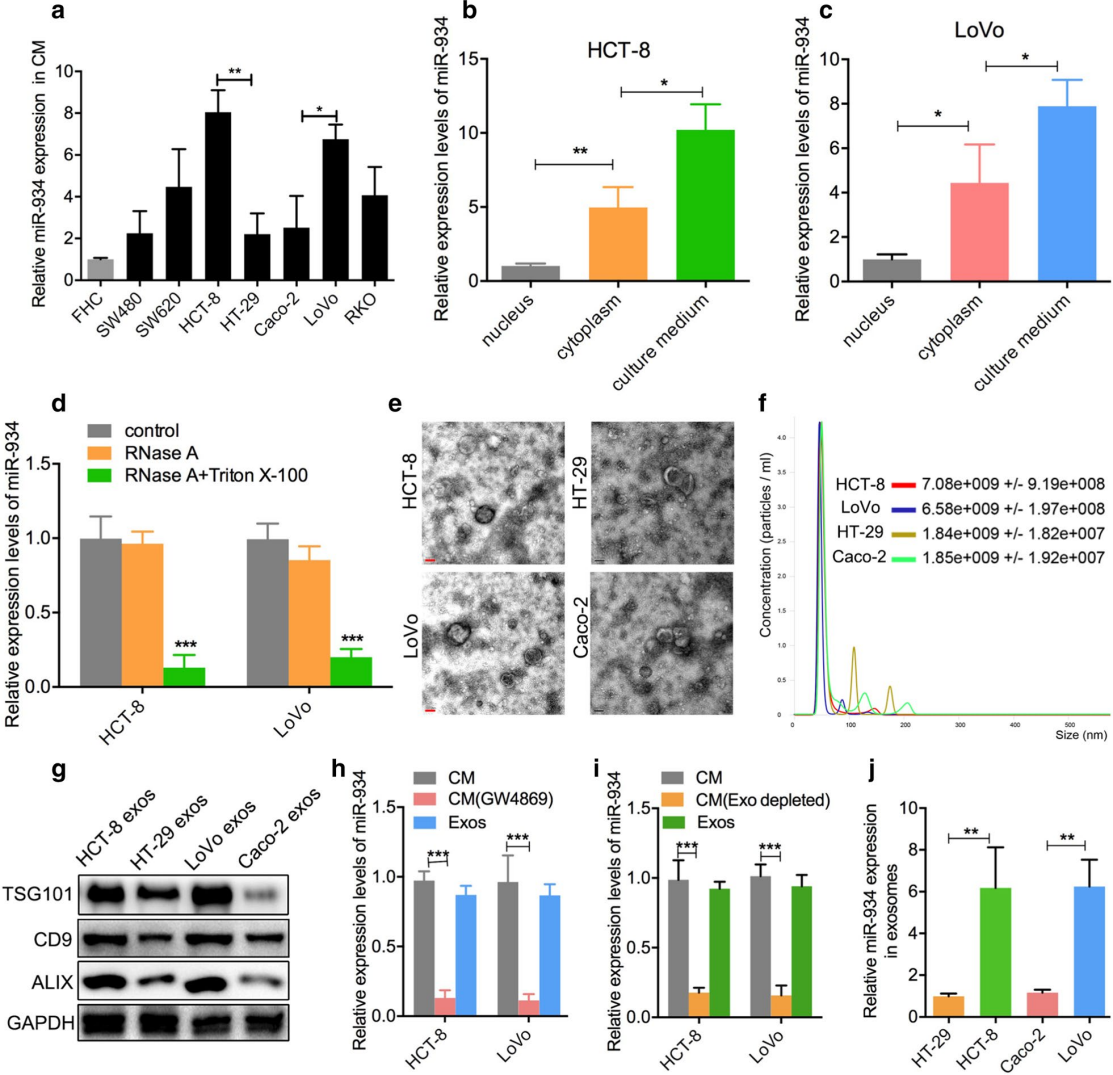
miR-934 is encapsulated within CRC cell-derived exosomes. **a** qPCR analysis of the expression levels of miR-934 in the culture medium (CM) of seven different CRC cell lines and one normal colon cell line. **b**, **c** qPCR analysis of the expression levels of miR-934 in the nucleus, cytoplasm, and culture medium (CM) of the HCT-8 and LoVo cell lines. These cell lines showed relatively higher miR-934 expression than other cell lines. **d** qPCR analysis of the expression levels of miR-934 in the HCT-8 and LoVo cell lines treated with control medium or RNase A (2 mg/mL) alone or in combination with Triton X-100 (0.1%), for 0.5 h. **e**, **f** Phenotype analysis of exosomes derived from HCT-8, HT29, LoVo, and Caco-2 cells using electron microscopy (**e**) and Nano Sight nanoparticle tracking analysis (**f**). **g** Western blot analysis was performed to detect typical exosomal biomarkers (TSG101, CD9, and ALIX) in exosomes derived from the above four CRC cell lines. **h**, **i** qPCR analysis of miR-934 expression in the CM of HCT-8/LoVo cells depleted of exosomes by GW4869 (an inhibitor of exosome secretion) (**h**) or by ultracentrifugation (**i**). **j** qPCR analysis of the expression levels of miR-934 in HT-29/HCT-8/LoVo/Caco-2-derived exosomes (*p < 0.05; **p < 0.01; ***p < 0.001)
