# Supplementary figures and images for: Tumor-derived exosomal miR-934 induces macrophage M2 polarization to promote liver metastasis of colorectal cancer
Source: J Hematol Oncol. 2020 Nov 19;13:156. doi: 10.1186/s13045-020-00991-2 (PMC7678301; doi:10.1186/s13045-020-00991-2)

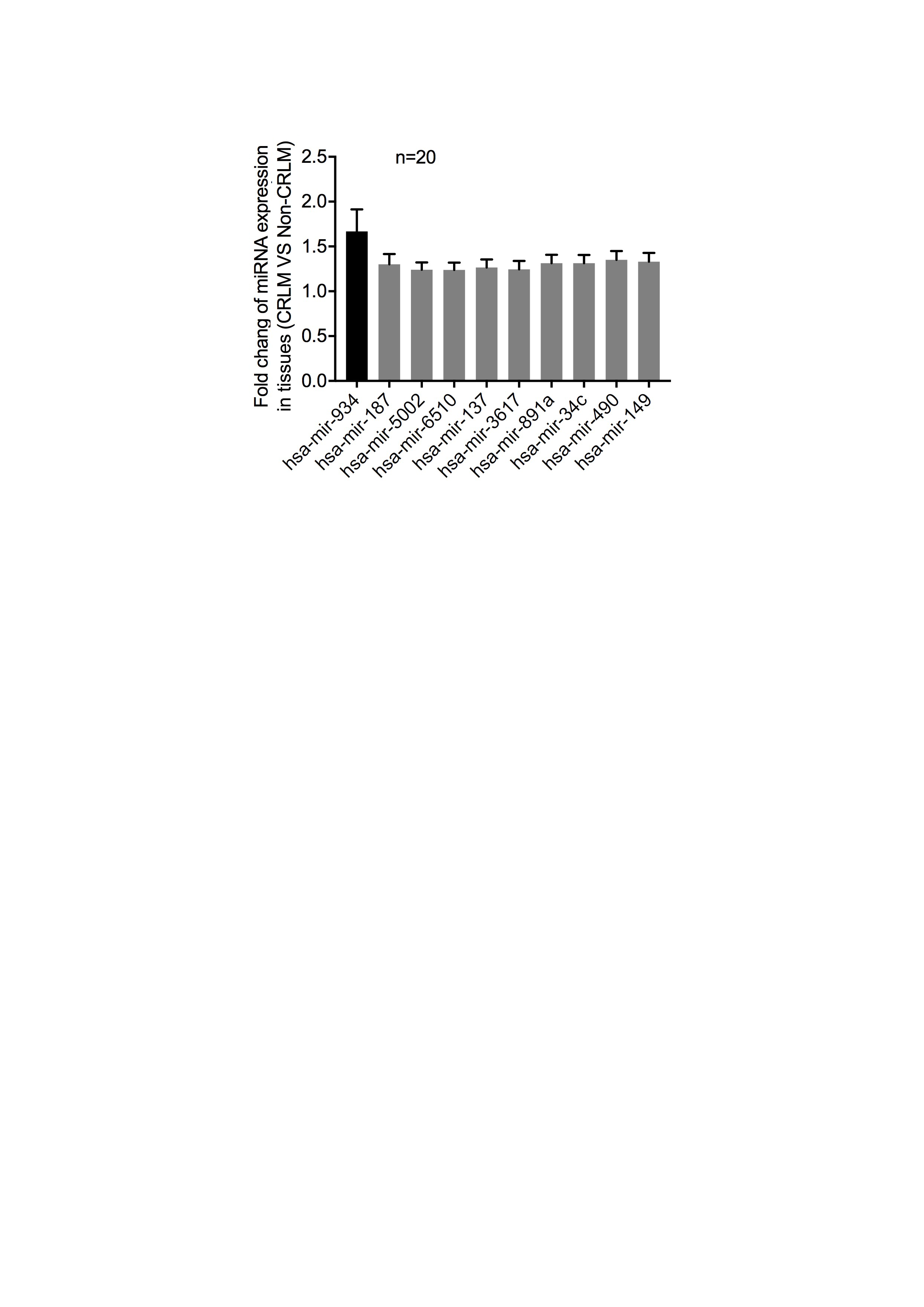

Supplement: Supplementary file 1 — Additional file 1: Figure S1. miR-934 is the top miRNA upregulated in CRLM compared to non-CRLM samples’ primary tumor tissues. qPCR analysis of the expression of the top ten upregulated miRNAs in 20 CRLM and 20 non-CRLM samples’ primary tumor tissues from the FUSCC database. [file 13045_2020_991_MOESM1_ESM.tif]

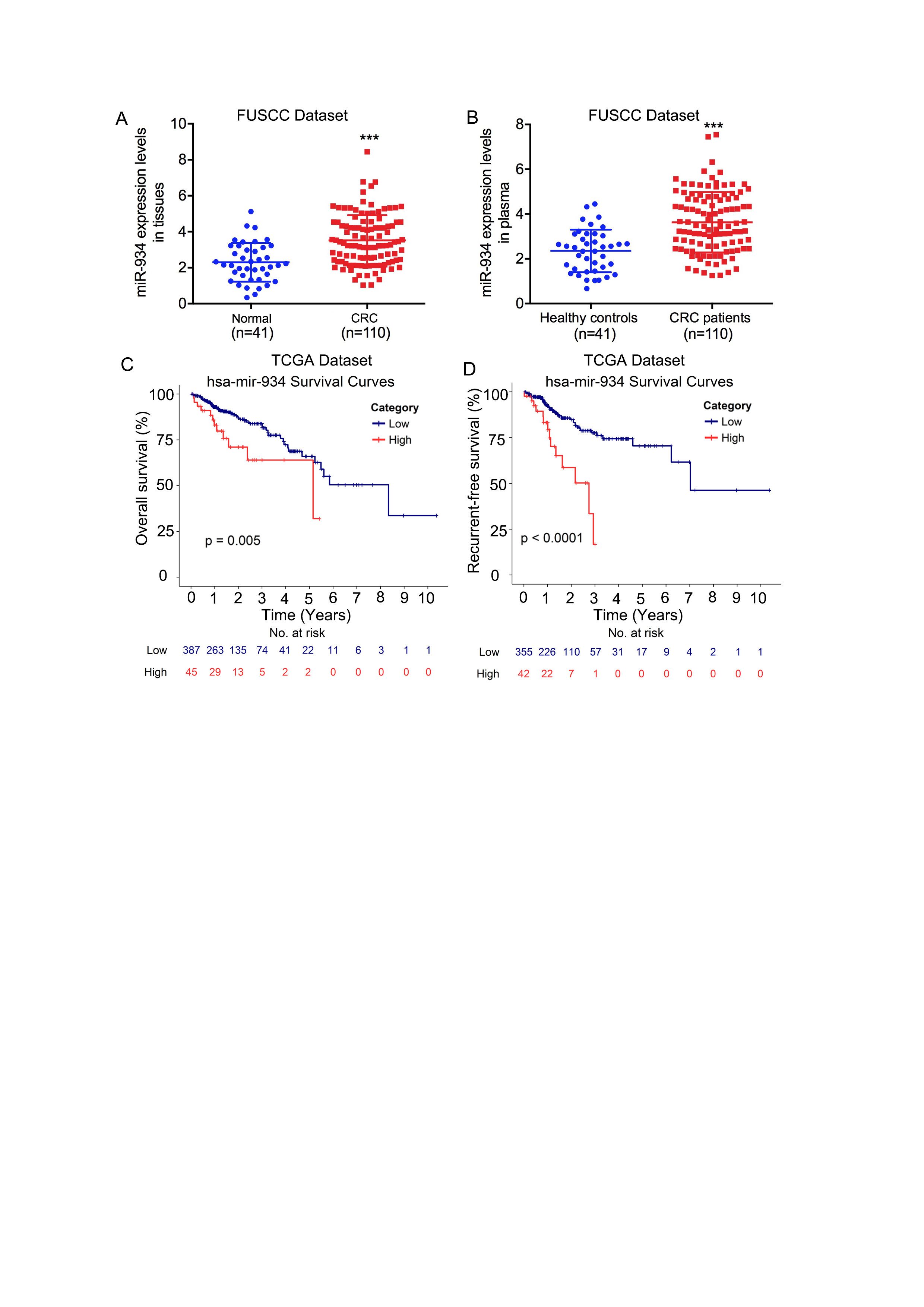

Supplement: Supplementary file 2 — Additional file 2: Figure S2. Levels of miR-934 in the tissues and serum of CRC patients from the FUSCC dataset and the role of miR-934 in predicting the OS and DFS of CRC patients from the TCGA dataset. a Expression of miR-934 in 41 normal tissues and 110 CRC tissues. b Expression of miR-934 in the serum of 41 healthy controls and 110 CRC patients. c, d. Kaplan–Meier survival analysis with the log-rank test was used to determine the association of miR-934 expression with the OS (c) and DFS (d) of CRC patients from the TCGA dataset (*p < 0.05; **p < 0.01; ***p < 0.001). [file 13045_2020_991_MOESM2_ESM.tif]

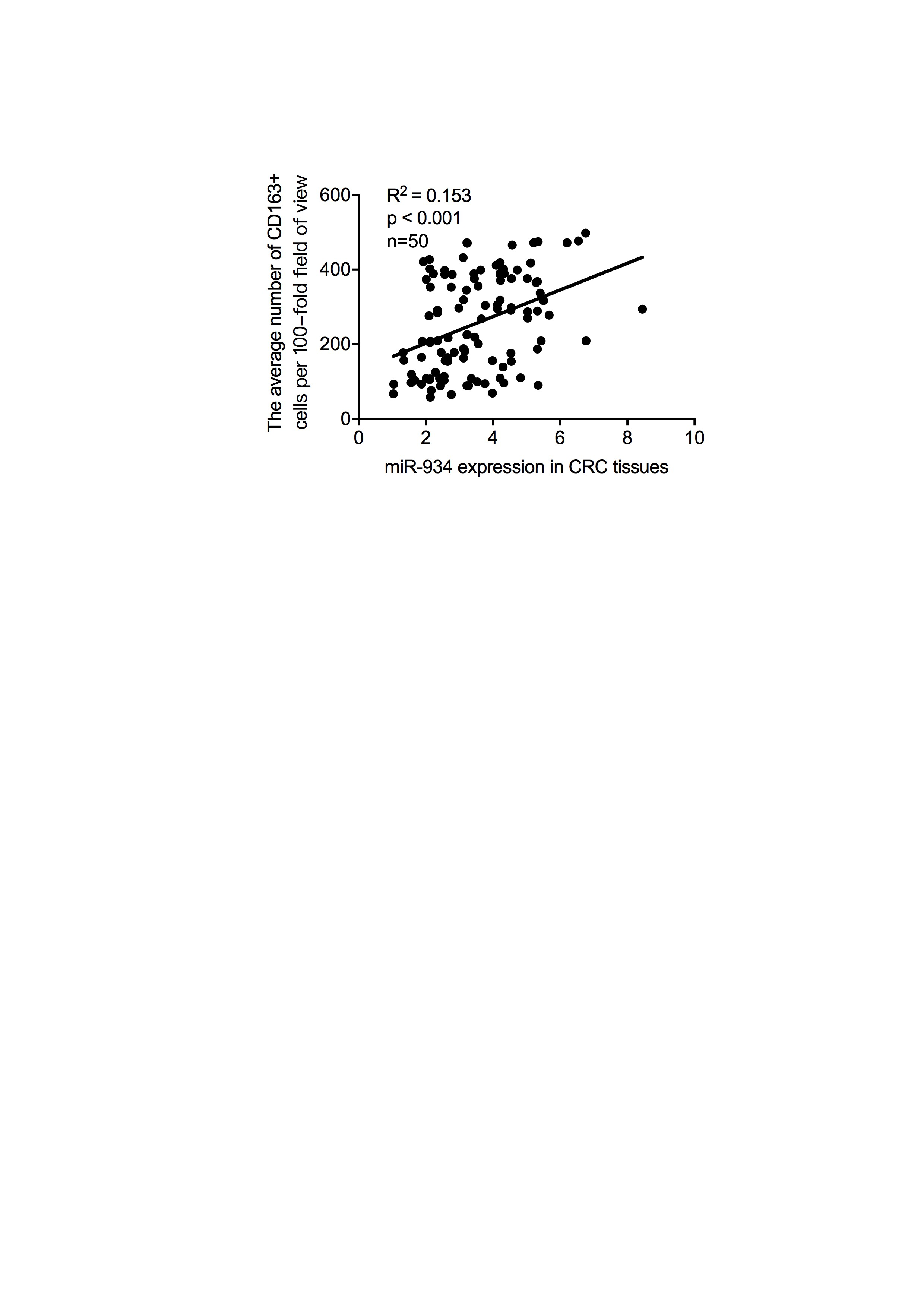

Supplement: Supplementary file 3 — Additional file 3: Figure S3. Spearman correlation analysis of the CD163 positivity rate and miR-934 expression in 50 CRC tissues. Spearman correlation analysis showed that the CD163 positivity rate was positively associated with miR-934 expression in 50 CRC tissues. [file 13045_2020_991_MOESM3_ESM.tif]

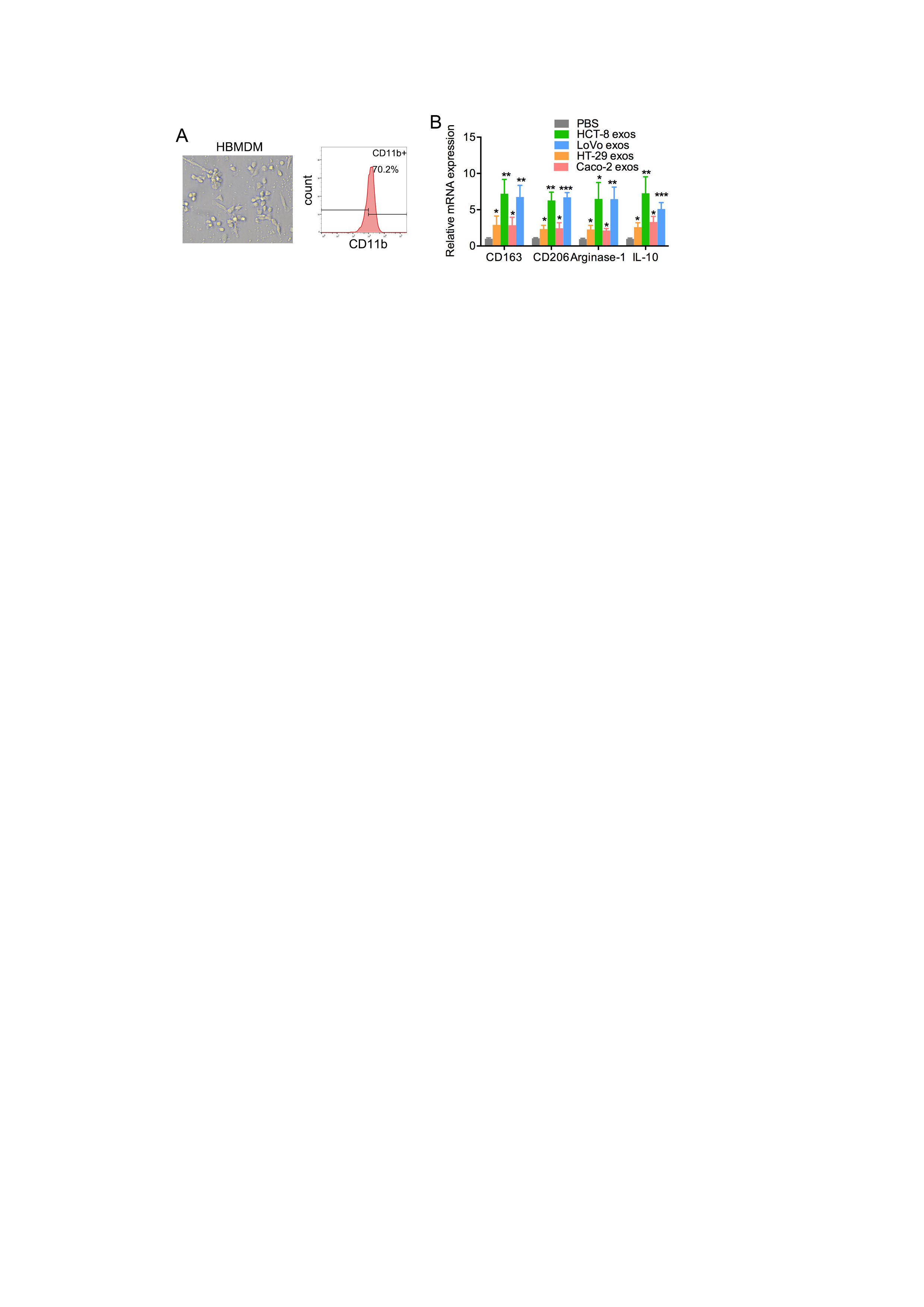

Supplement: Supplementary file 4 — Additional file 4: Figure S4. Effects of exosomal miR-934 on the polarization of HBMDMs. a Morphology of human bone marrow-derived macrophages (HBMDMs). The macrophage marker CD11b was measured with flow cytometry. b qPCR analysis of the changes in M2 marker (CD163, CD206, Arginase-1, and IL-10) expression levels after HBMDMs were treated with CRC cell-derived exosomes (*p < 0.05; **p < 0.01; ***p < 0.001). [file 13045_2020_991_MOESM4_ESM.tif]

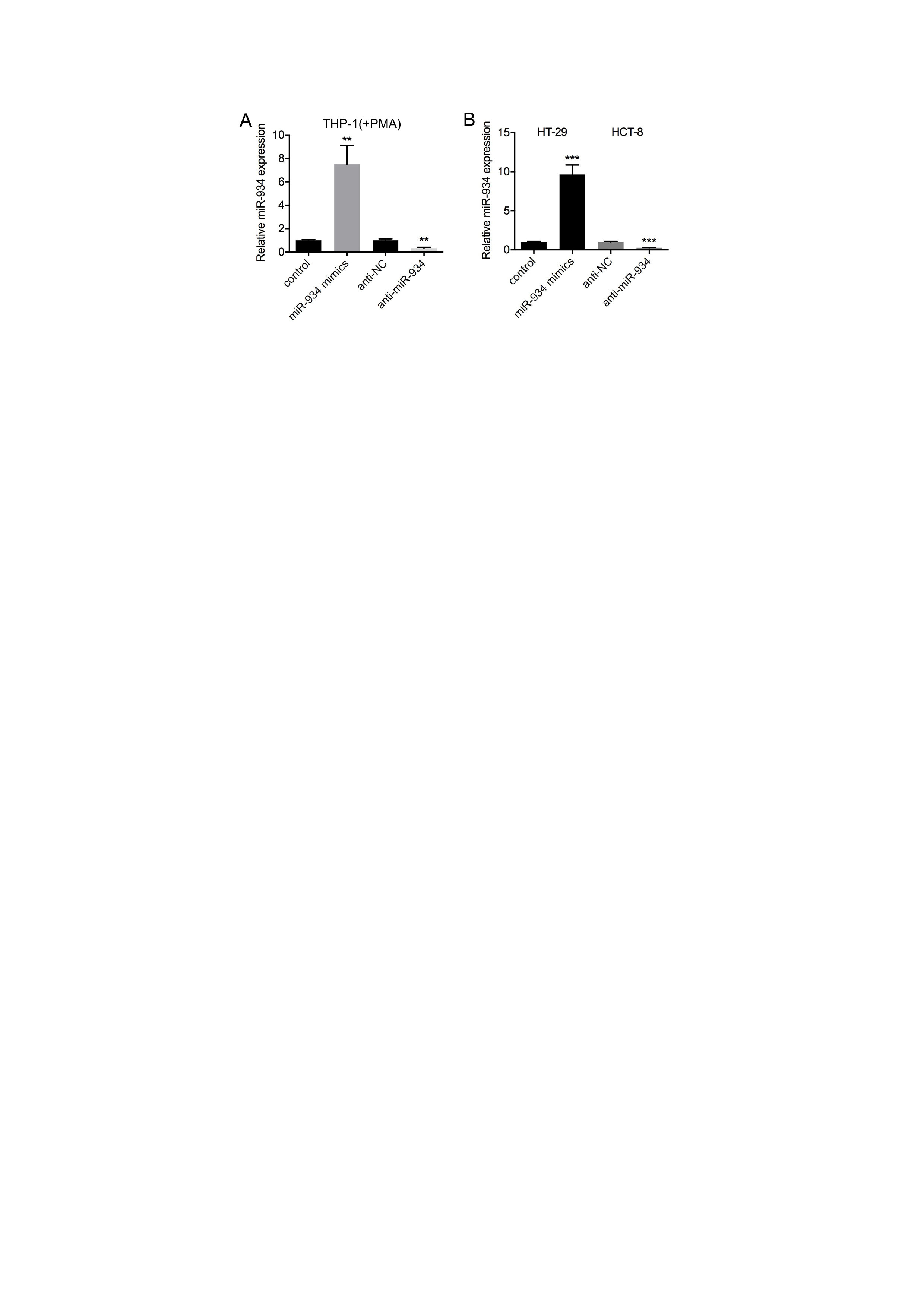

Supplement: Supplementary file 5 — Additional file 5: Figure S5. Effect of miR-934 mimics and anti-miR-934 vectors on THP-1 cells prestimulated with PMA and CRC cells. a Levels of miR-934 in THP-1 cells prestimulated with PMA and transfected with miR-934 mimics, anti-miR-934 vectors, or their control vectors. b Levels of miR-934 in HT-29 and HCT8 cells transfected with miR-934 mimics, anti-miR-934 vectors, or their control vectors (**p < 0.01; ***p < 0.001). [file 13045_2020_991_MOESM5_ESM.tif]

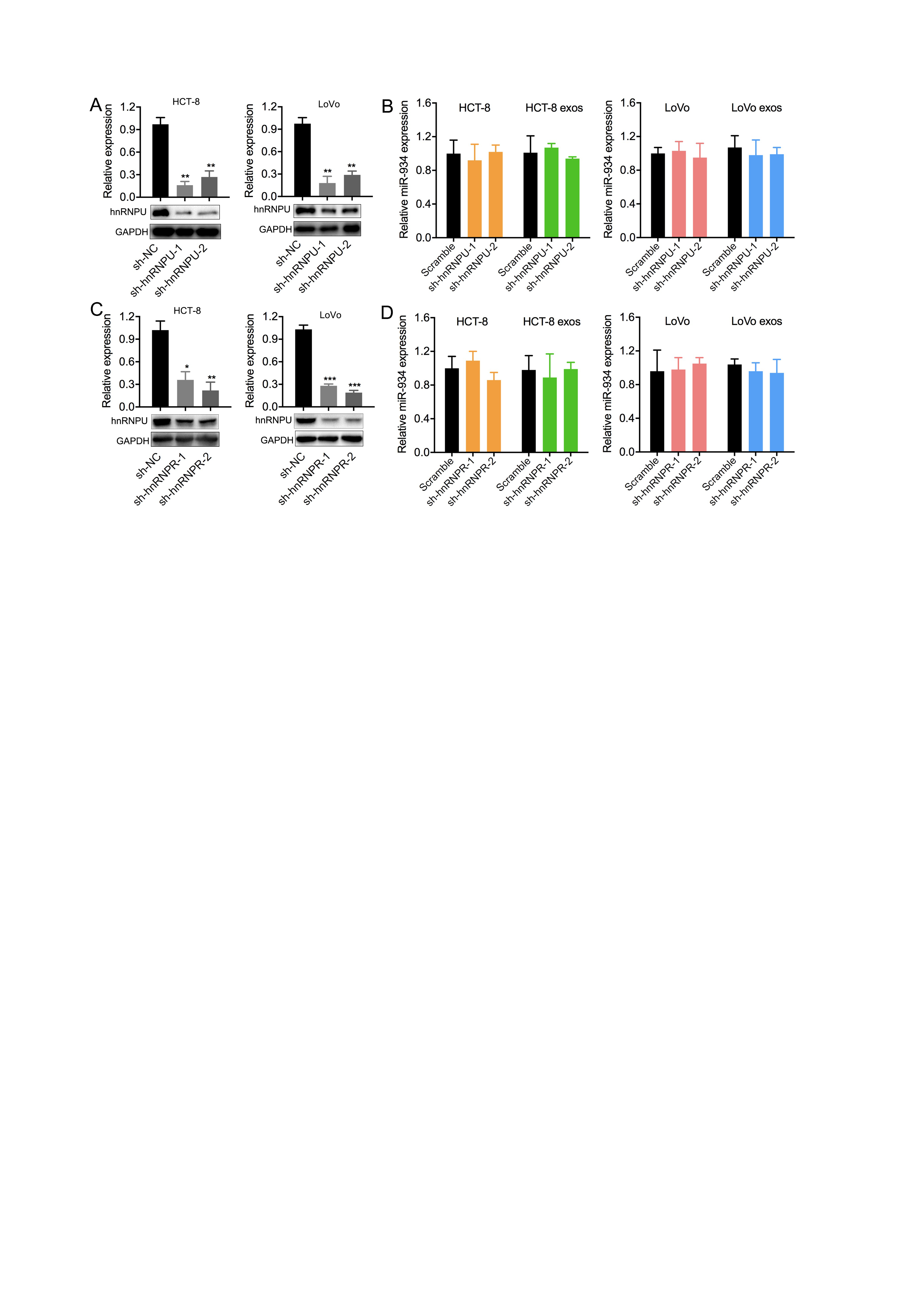

Supplement: Supplementary file 6 — Additional file 6: Figure S6. Effect of the RNA binding proteins hnRNPU and hnRNPR on the expression of CRC cell-derived exosomal miR-934. a, b Changes in the mRNA and protein expression levels of hnRNPU and hnRNPR induced by transfection of their knockdown plasmids. c, d Levels of total and exosomal miR-934 after transfection of shhnRNPU, shhnRNPR, or their negative control plasmids into HCT8 and LoVo cells (*p < 0.05; **p < 0.01; ***p < 0.001). [file 13045_2020_991_MOESM6_ESM.tif]

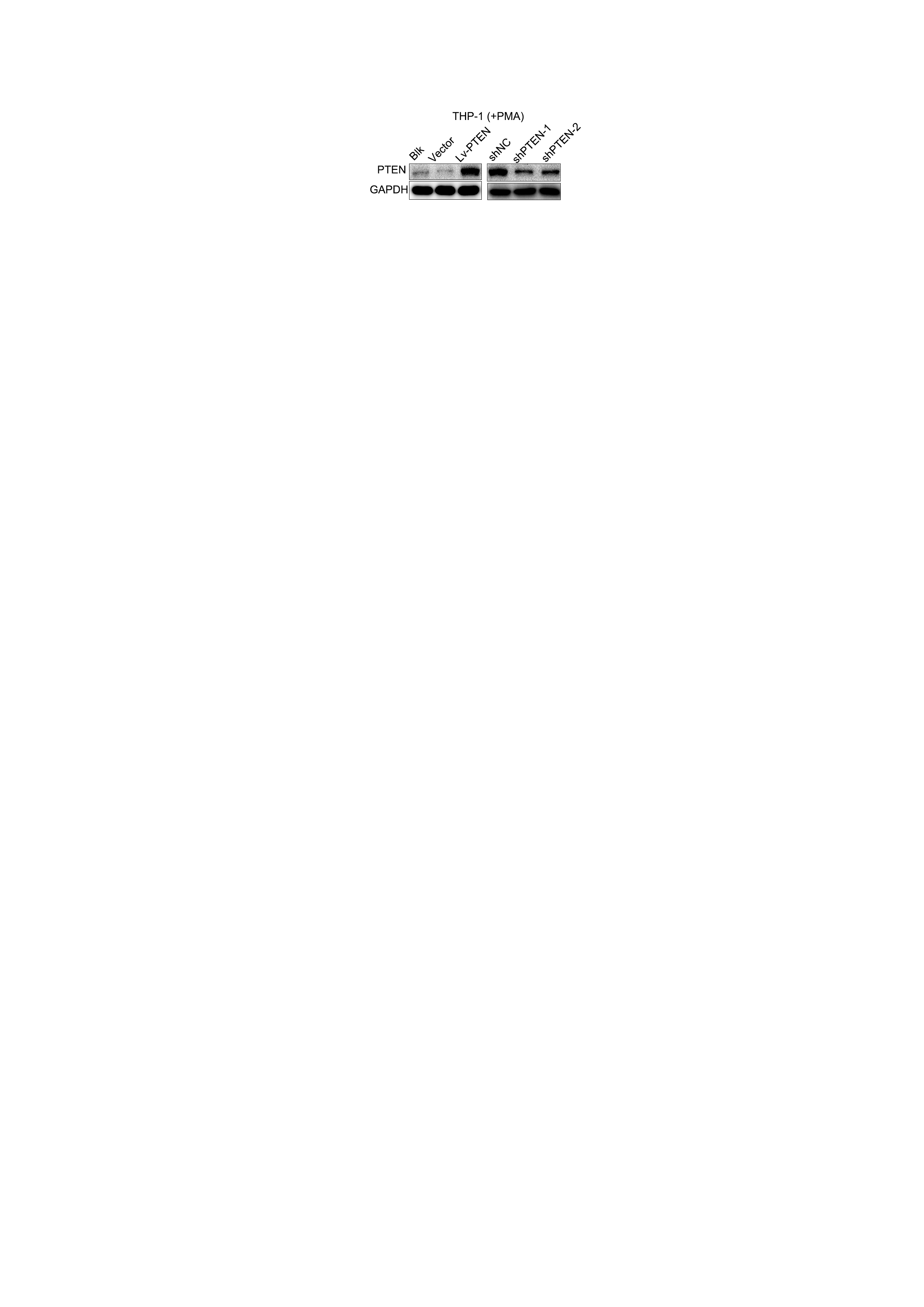

Supplement: Supplementary file 7 — Additional file 7: Figure S7. Changes in the expression of PTEN induced by transfection of overexpression (a) or knockdown (b) vectors into PMA-treated THP-1 cells. [file 13045_2020_991_MOESM7_ESM.tif]

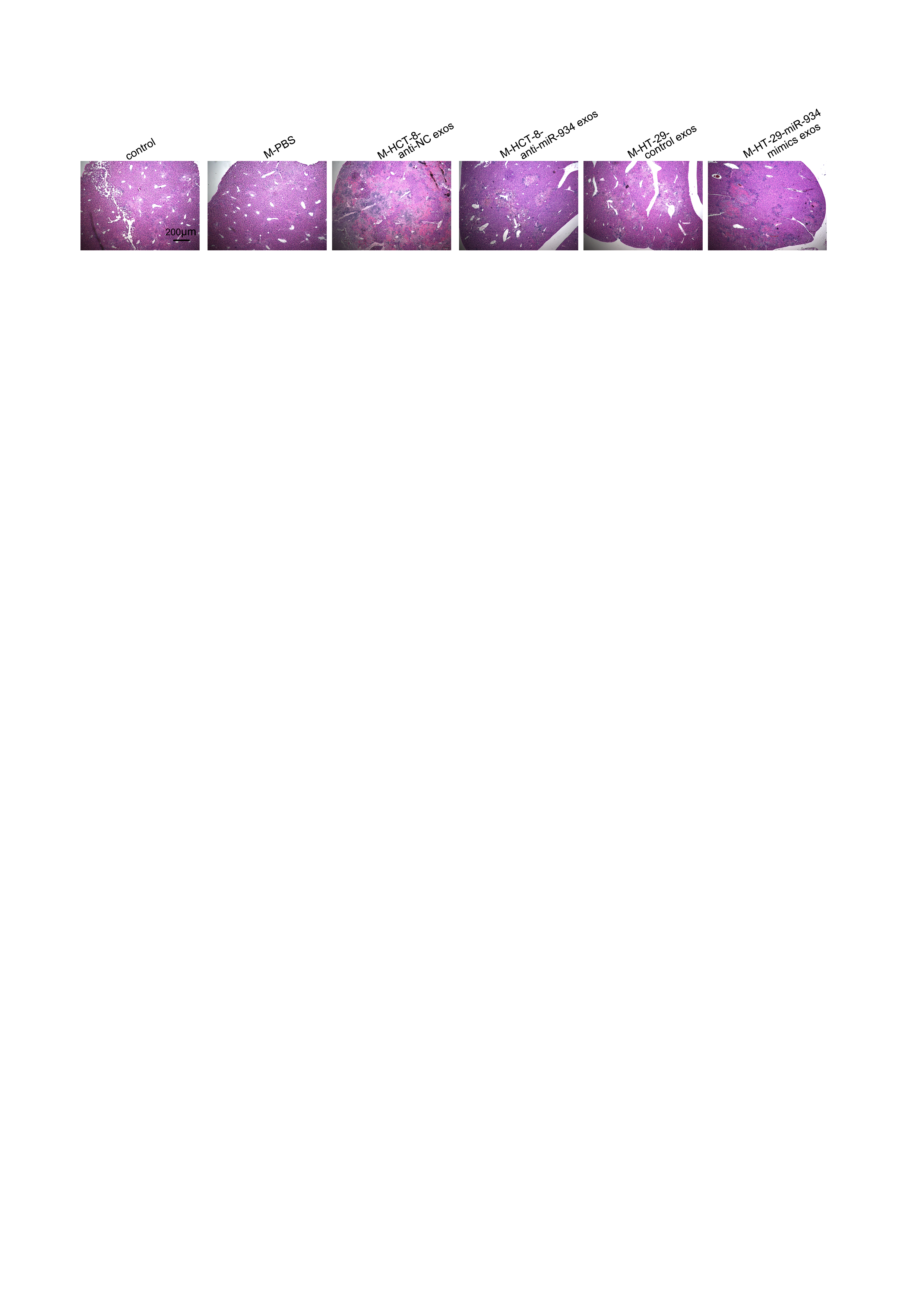

Supplement: Supplementary file 8 — Additional file 8: Figure S8. Representative HE staining images of each group (as a supplement to Fig. 6e). [file 13045_2020_991_MOESM8_ESM.tif]

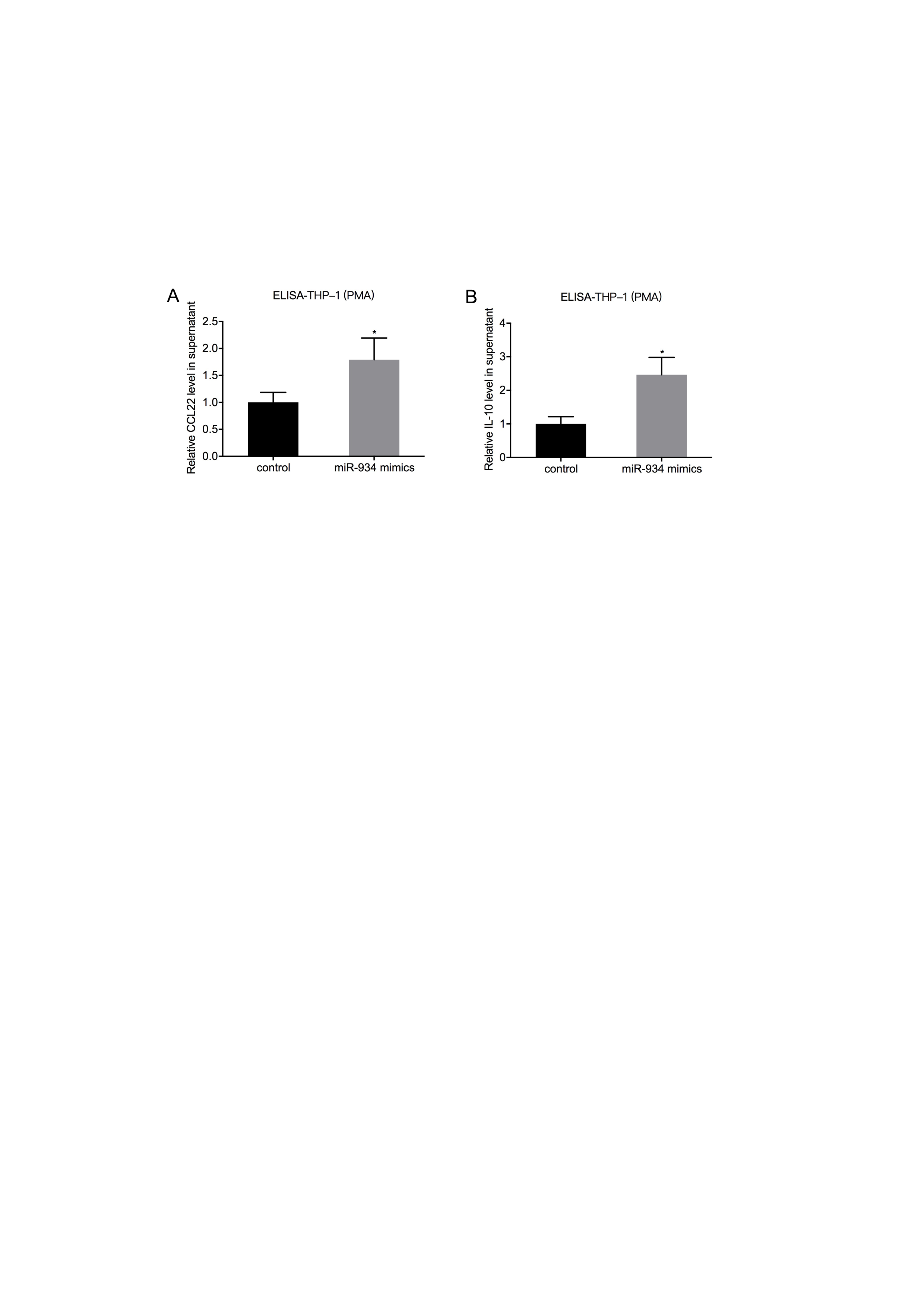

Supplement: Supplementary file 9 — Additional file 9: Figure S9. Changes in the secretion of CCL22 and IL-10 after THP-1 cells prestimulated with PMA were transfected with miR-934 mimics. ELISA assays examined CCL22 (a) and IL-10 (b) in the CM of THP-1 cells prestimulated with PMA and transfected with miR-934 mimics (*p < 0.05). [file 13045_2020_991_MOESM9_ESM.tif]

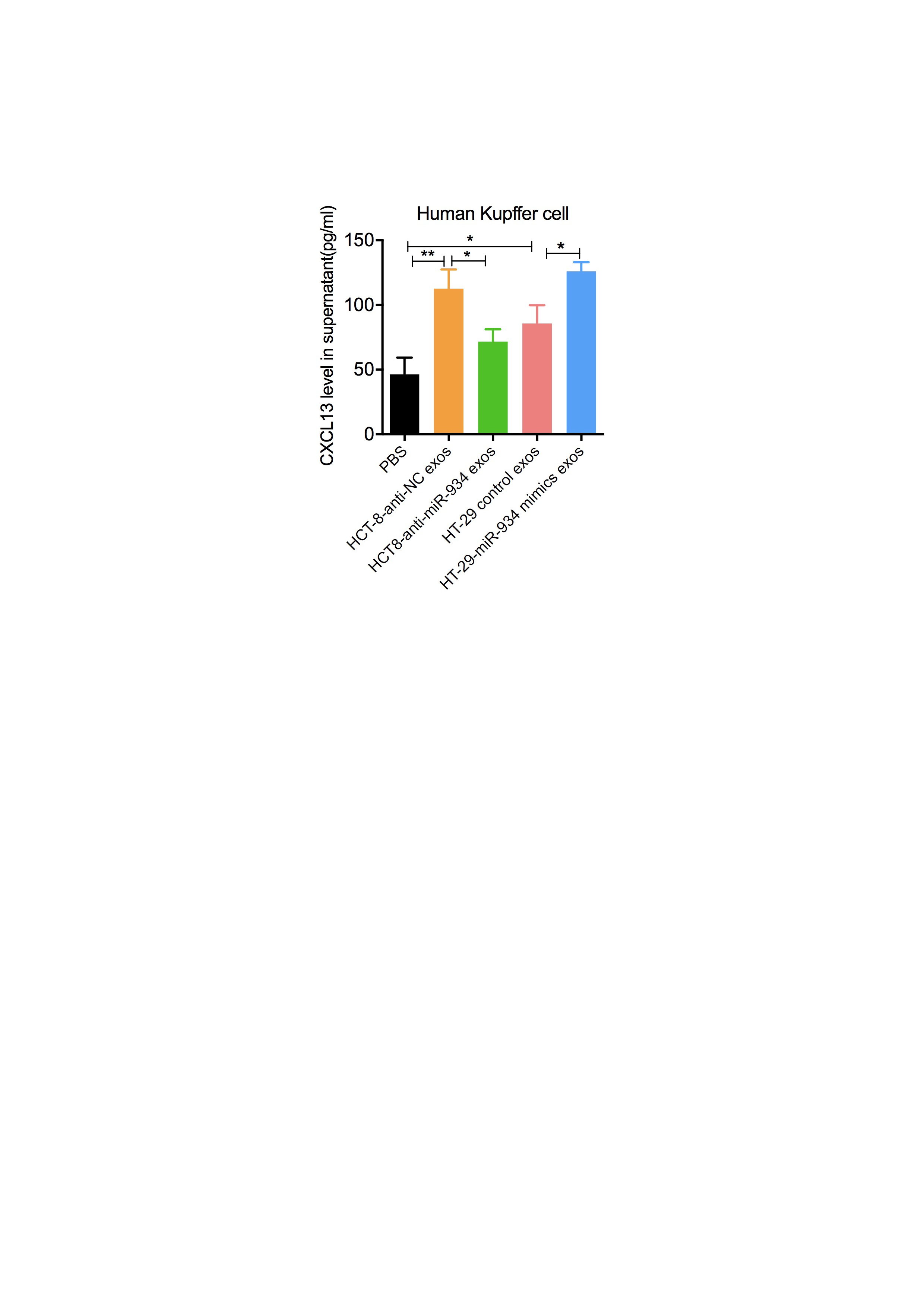

Supplement: Supplementary file 10 — Additional file 10: Figure S10. Effects of exosomal miR-934 on the secretion of CXCL13 by Kupffer cells. ELISA measuring CXCL13 in the CM of human Kupffer cells pretreated with exosomes derived from HCT-8/HT-29 cells and transfected with anti-miR-934 or miR-934 mimics (*p < 0.05; **p < 0.01). [file 13045_2020_991_MOESM10_ESM.tif]

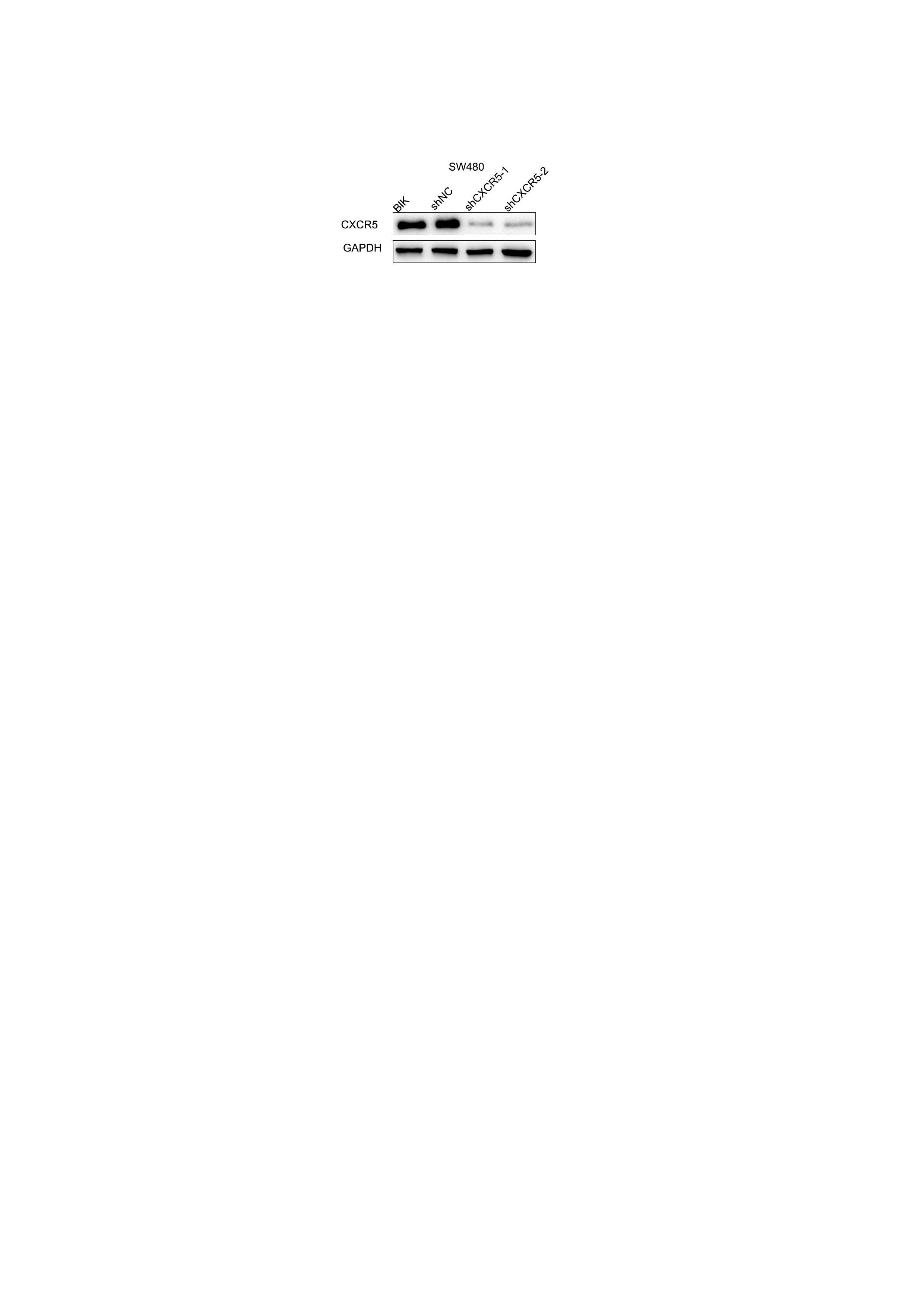

Supplement: Supplementary file 11 — Additional file 11: Figure S11. Changes in the expression of CXCR5 induced by transfection of its knockdown vectors into SW480 cells. [file 13045_2020_991_MOESM11_ESM.tif]

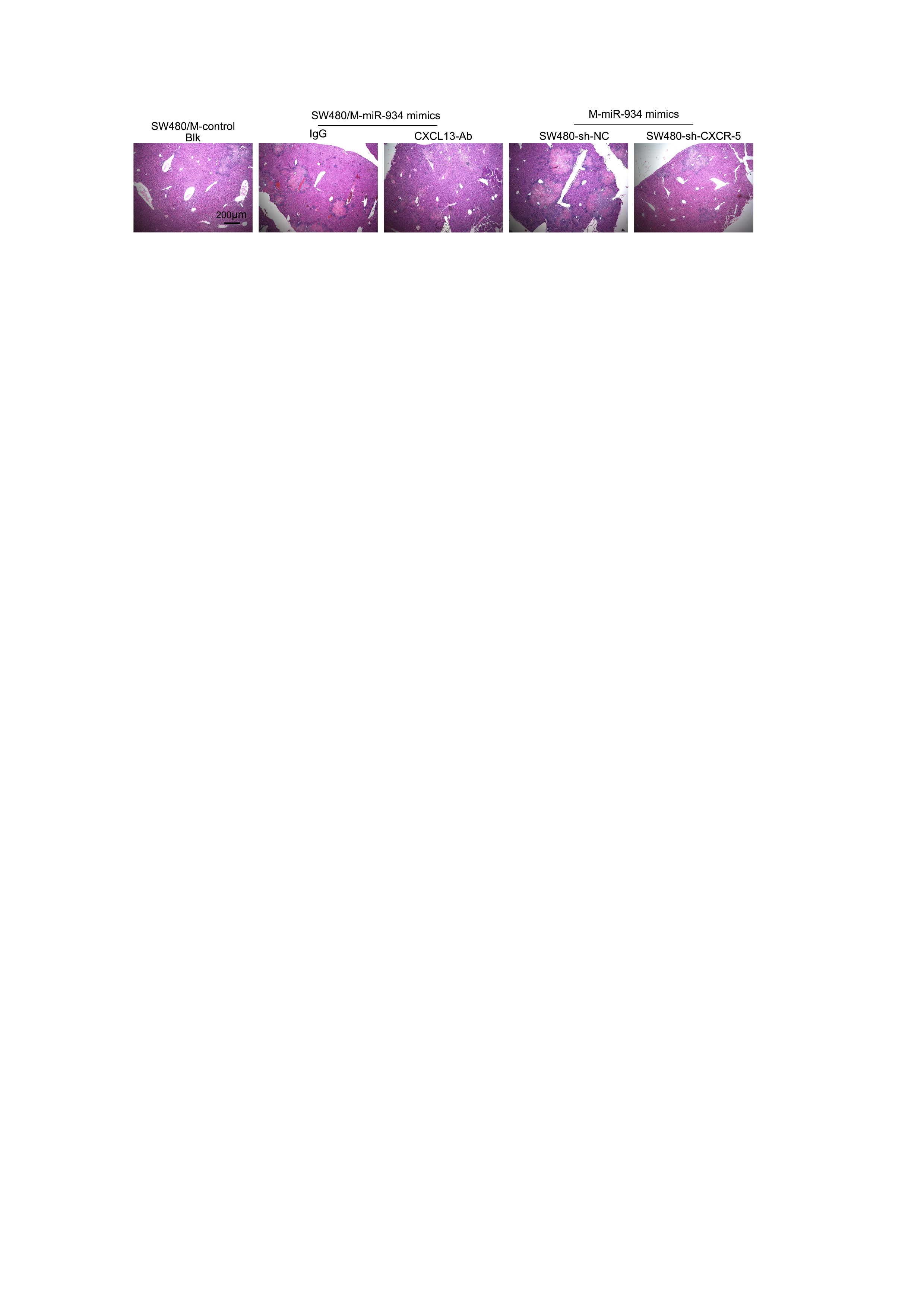

Supplement: Supplementary file 12 — Additional file 12: Figure S12. Representative HE staining images of each group (as a supplement to Fig. 7g). [file 13045_2020_991_MOESM12_ESM.tif]

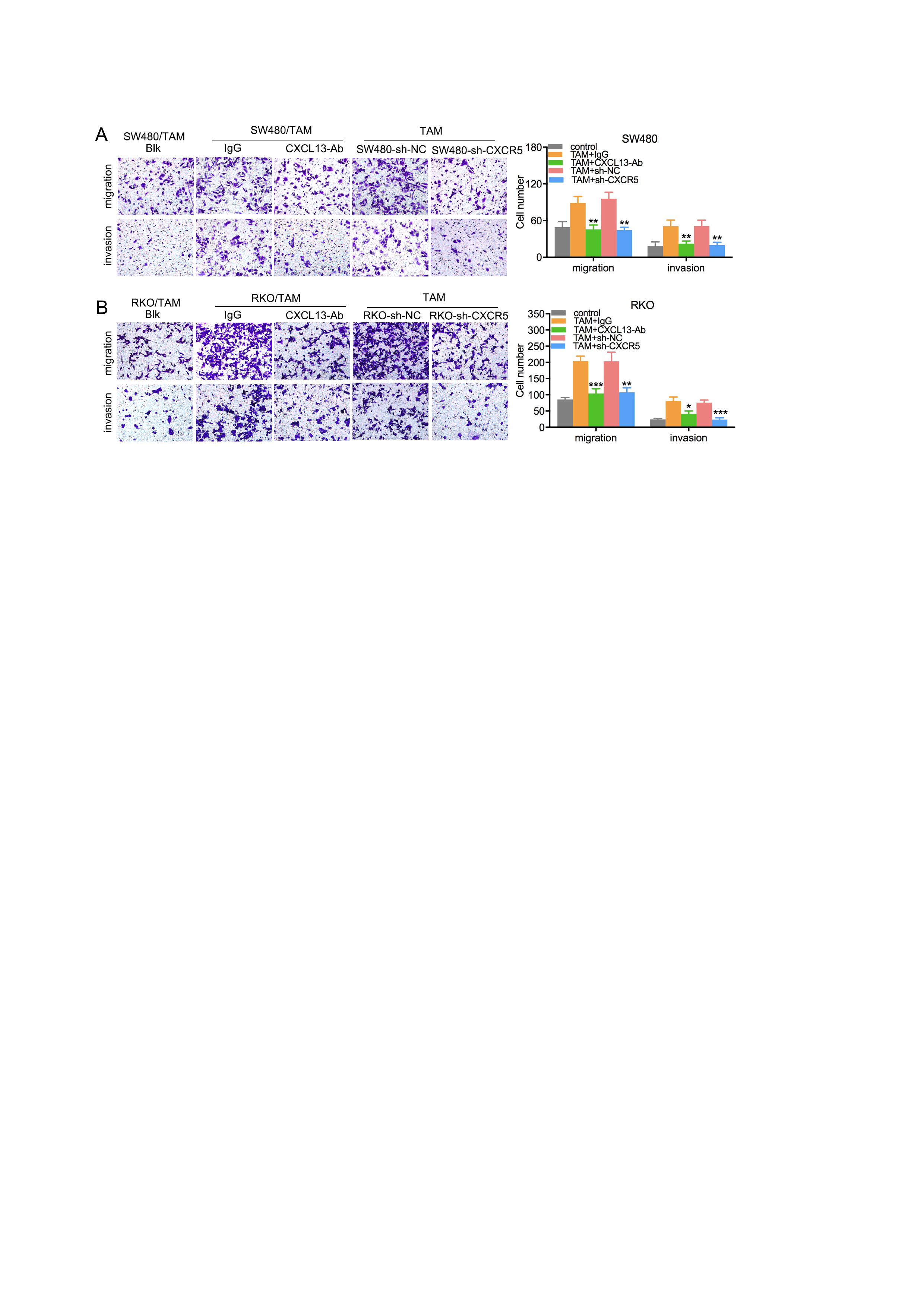

Supplement: Supplementary file 13 — Additional file 13: Figure S13. TAMs promote the migration and invasion of CRC cells by activating the CXCL13/CXCR5 axis. a, b TAMs were cocultured with CRC cells and anti-CXCL13 antibody or cocultured with CRC cells transfected with sh-CXCR5. Migration and invasion in vitro were evaluated using a transwell assay (*p < 0.05; **p < 0.01; ***p < 0.001). [file 13045_2020_991_MOESM13_ESM.tif]

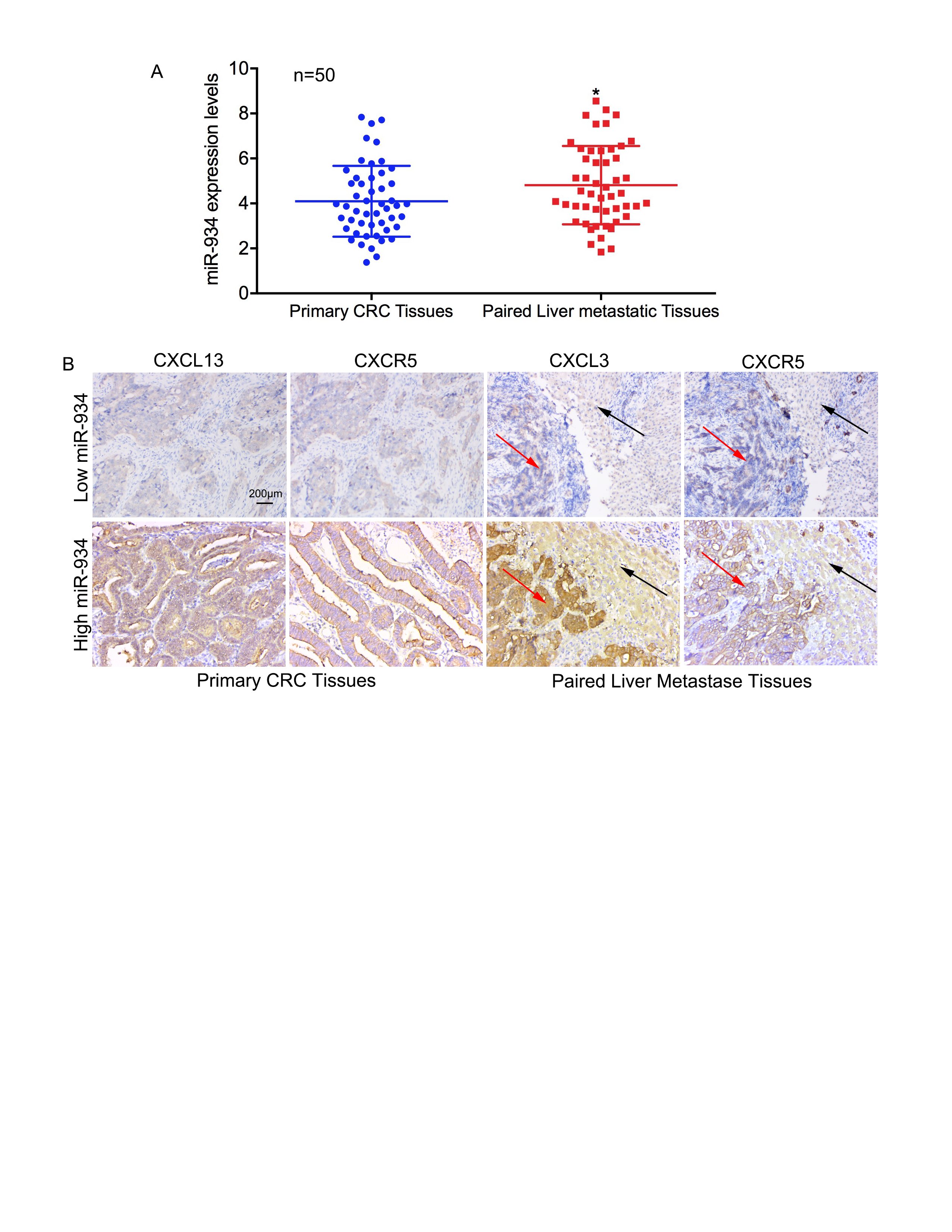

Supplement: Supplementary file 14 — Additional file 14: Figure S14. Association of miR-934 expression in CRC tissues and paired liver metastatic tissues with CXCL13 and CXCR5 expression in CRC tissues, adjacent normal liver tissues and paired liver metastatic tissues. a A qPCR assay was used to examine miR-934 expression in 50 CRC tissues and paired liver metastatic tissues. b Representative images of IHC staining of CXCL13 and CXCR5 in 50 CRC tissues, adjacent normal liver tissues and paired liver metastatic tissues (*p < 0.05; The red arrows indicate paired liver metastatic tissues; the black arrows indicate adjacent normal liver tissues; Scale bar, 200 μm). [file 13045_2020_991_MOESM14_ESM.tif]
