# Supplementary material for: Tumor-derived exosomal miR-934 induces macrophage M2 polarization to promote liver metastasis of colorectal cancer
Source: J Hematol Oncol. 2020 Nov 19;13:156. doi: 10.1186/s13045-020-00991-2 (PMC7678301; doi:10.1186/s13045-020-00991-2)
Supplement: Supplementary file 15 — Additional file 15: Supplementary materials and methods. [file 13045_2020_991_MOESM15_ESM.docx]

**Supplementary materials and methods**

**Tissue microarray (****TMA) construction and *in situ* hybridization (ISH) assay**

A total of 308 paired CRC tissues and adjacent normal mucosa samples were used for construction of a TMA. TMA construction and ISH were performed according to our previous study.^(1)^

**Cell culture**

The human CRC cell lines HCT-8, LoVo, Caco-2, HT-29, SW480, SW620, and RKO and the human monocytic cell line THP-1 were obtained from Type Culture Collection of Chinese Academy of Sciences (Shanghai, China). Human bone marrow-derived macrophages (HBMDMs) were isolated from human bone marrow. To induce differentiation into macrophages, THP-1 cells (1×10^6^) were treated with 100 ng/mL phorbol 12-myristate 13-acetate (PMA; Abcam, UK) for 48 h. All cells were maintained at 37 ℃ in a 5% CO_2_ humidified atmosphere and cultured in Dulbecco’s modified Eagle medium (DMEM; Thermo Fisher Scientific, USA) containing 10% fetal bovine serum (FBS; Thermo Fisher Scientific, USA). All cell lines were tested using short tandem repeat analysis, and the last authentication was performed in March 2019.

**Cell transfection**

Restoration or inhibition of miR-934 expression was achieved by transfecting cells with miR-934 mimics or anti-miR-934 vectors, which were purchased from GenePharma (Shanghai, China). Overexpression or silencing of phosphatase and tensin homolog (PTEN), heterogeneous nuclear ribonucleoprotein A2/B1 (hnRNPA2B1) and C-X-C motif receptor 5 (CXCR5) was achieved by transfecting cells with the respective Lv-PTEN, shPTEN, shhnRNPA2B1or shCXCR5 plasmids. Empty mimics (miR-NC), anti-control (Anti-Ctrl), empty vector (Vector), and scramble were used as controls for miR-934 mimics, anti-miR-934, Lv-PTEN, shPTEN, shhnRNPA2B1 and shCXCR5. Cell transfection was performed as previously described. ^(2)^ All sequences used in this study are summarized in Supplementary Table S1.

**Isolation and analysis of exosomes**

For exosome isolation, supernatant collected from 3-day cell cultures was first centrifuged at 500 g for 10 min to remove any cell contamination. Next, the upper supernatant was further centrifuged at 12,000 g for 20 min to remove any possible apoptotic bodies and large cell debris. Final centrifugation was performed at 100,000 g for 70 min to enrich exosomes, and the pellet was rinsed in 20 mL of phosphate-buffered saline (PBS). Finally, exosomes were collected by ultracentrifugation at 100,000 g for 70 min. The number and morphology (cup-shaped) of exosomes were examined using a NanoSight NS300 microscope (Malvern Instruments Ltd., UK) and a Philips CM120 BioTwin transmission electron microscope (FEI Company, USA), respectively.

**Fluorescent labeling and tracing of exosomal miR-934**

The CRC cell lines HCT-8 and HT29 were pretreated with FITC-labeled miR-934. Next, the CRC cells were cocultured with THP-1 cells for 48 h in 24-well transwell chambers. Internalization of exosomal miR-934 was examined using a TCS SP8 confocal laser-scanning microscope (Leica, Germany). The nuclei of THP-1 cells were stained with 4′,6-diamidino-2-phenylindole (DAPI; Roche, Switzerland).

**Quantitative PCR (qPCR)****, western blotting (WB), hematoxylin–eosin staining, (HE) and immunohistochemical (IHC) staining**

qPCR, WB, HE staining, and IHC staining were performed as described previously. ^(1,2)^ U6 and GAPDH served as internal controls for qPCR and WB, respectively. In 110 fresh tumor tissues, we calculated the average expression level of miR934 based on qPCR data. We defined high and low levels of miR-934 according to whether the expression value was higher or lower than the average level. Nuclear RNA and cytoplasmic RNA were extracted with a Cytoplasmic and Nuclear RNA Purification Kit (NORGEN). miRNA in culture medium was extracted using the mirVana™ PARIS™ Kit (Ambion, USA). The synthesized exogenous reference cel-miR-39 (1 pmol per sample; TIANGEN, China) was added to each RNA sample (50 μL). After reverse transcription, a qPCR assay was performed, and the relative miR-934 expression levels in the nucleus, cytoplasm and culture media were normalized against those of the exogenous reference cel-miR-39. The percentage of positive cells in the IHC assay was scored as follows: 1) staining area score: 0, < 10%; 1, 10–30%; 2, 31–50%; and 3, > 50%; 2) staining intensity score: 0, no staining; 1, mild staining; 2, moderate staining; and 3, intense staining; 3) total staining score based on both staining area and intensity: 0–2, negative expression; 3–4, medium expression; and 5–6, strong expression. All antibodies used in this study are summarized in Supplementary Table S2.

**Flow cytometry**

THP-1 cells were harvested, washed with PBS, and fixed with 1% paraformaldehyde (PFA) overnight at 4 °C. Following overnight incubation, the cells were washed again, resuspended in ﬂow cytometry buffer (1× PBS buffer containing 1% FSA), and stained with anti-CD163 and anti-CD11B antibodies (#[562643](https://www.bdbiosciences.com/cn/reagents/research/antibodies-buffers/immunology-reagents/anti-human-antibodies/cell-surface-antigens/bv421-mouse-anti-human-cd163-ghi61/p/562643) and #[562399](https://www.bdbiosciences.com/cn/applications/research/stem-cell-research/mesenchymal-stem-cell-markers-bone-marrow/human/negative-markers/pe-cf594-mouse-anti-human-cd11b-icrf44-also-known-as-44/p/562399), respectively, BD Biosciences, USA) for 30 min at 20℃. Finally, the cells were washed, resuspended, and analyzed using flow cytometry (BD Biosciences, USA) according to the manufacturer’s instructions.

**Biotin miRNA pull-down assay**

Biotin-labeled wild-type and mutant miR-934 were synthesized by GenePharma (China) and transfected into CRC cell lines (HCT-8 and LoVo) at 20 ℃ for 48 h. In accordance with the manufacturer's instructions, cytoplasmic and nuclear extracts were acquired using NE-PERTM nuclear and cytoplasmic extraction reagents (Thermo Fisher Scientific, USA). Subsequently, nuclear, cytoplasmic, or exosomal extracts of HCT-8 and LoVo cells were incubated overnight at 4 ℃, following incubation with M-280 streptavidin magnetic beads (Sigma-Aldrich, USA). Finally, the precipitates were washed and analyzed using WB; biotinylated poly(G) (5′-GGGGGGGGGGGGGGGGGGGGG-3′) served as the negative control.

**RNA-binding protein immunoprecipitation (RIP) assay**

RIP assays were performed using the Magna RIP™ Kit (Millipore, USA) according to the manufacturer’s instructions. Briefly, cells were lysed in the presence of protease inhibitors and RNase inhibitors. The protein extract was incubated with anti-hnRNPA2B1 antibody (Abcam, USA) or anti-IgG antibody (negative control) (Proteintech, USA). Next, A/G protein magnetic beads were added to the protein extract; the beads were incubated and washed, followed by extraction of coimmunoprecipitated miRNAs using the mirVana™ PARIS™ Kit (Ambion, USA). Next, the coimmunoprecipitated miRNAs were isolated, reverse transcribed, and analyzed using qPCR. Fold changes in miRNA enrichment in the immunoprecipitated samples are presented as percent input.

**Luciferase reporter assay**

To generate the luciferase construct, the PTEN gene, which contains miR-934 binding sites, was synthesized and cloned into GV272 vectors by GeneChem (China). Next, PMA-treated THP-1 cells were cotransfected with PTEN–3′-UTR–Luc fireﬂy luciferase constructs (wild-type or mutant) and miR-934 mimics or anti-miR-934 using Lipofectamine 2000 reagent (Invitrogen, USA). Finally, 48 h post transfection, cell lysates were harvested, and the firefly/Renilla luciferase activities were measured according to the Dual-Luciferase Reporter Assay Kit protocol (Promega, USA) using a Veritas™ 96-well Microplate Luminometer (Promega, USA) with a substrate dispenser (Promega, USA). Renilla luciferase activity was normalized against firefly luciferase activity.

**Transwell assay**

Tumor cell migration and invasion were examined using a transwell assay. According to the manufacturer’s protocol, a Transwell 24-well Boyden chamber (Corning, USA) with an 8.0-μm pore size polycarbonate membrane was used for cell migration (without Matrigel) and invasion (with Matrigel) assays. Briefly, 5 ×10^4^ tumor cells suspended in 200 µL of serum-free medium were seeded in the top chamber, while the bottom chambers contained 600 µL of medium supplemented with 10% FBS. After incubation at 37 °C in a 5% CO_2_ humidified atmosphere for 48 h, the migrated or invaded cells in the bottom chamber were fixed with methanol and stained with 0.1% crystal violet.

**Animal models**

To investigate the metastatic effect of CRC-derived exosomes *in vivo*, we established a liver metastasis model using four-week-old male, specific pathogen-free BALB/c nude mice. Each group contained five mice, and all mice were housed in a specific pathogen-free environment in the Animal Laboratory Unit. A total of 1×10^6^ luciferase-labeled CRC cells were cotransfected with conditioned medium and injected into the spleens of nude mice. After six weeks, anesthetized mice were [intraperitoneal](javascript:;)ly [injected](javascript:;) with D-luciferin (150 mg/kg) and imaged 10 min after injection using the IVIS Illumina System (Caliper Life Sciences, USA). Finally, the mice were euthanized, and liver samples were collected, embedded in paraffin, and subjected to HE staining. All animal experiments were approved by the Institutional Animal Care and Use Committee of Fudan University Shanghai Cancer Center.

**Chromatin immunoprecipitation (ChIP) assay**

ChIP assays were performed using an EZ-ChIP assay kit (Millipore, USA) according to the manufacturer's instructions. Briefly, cells (1×10^7^ per assay) were collected, cross-linked with 1% formaldehyde, quenched in glycine, lysed by sonication in the presence of protease inhibitors, and immunoprecipitated with anti-p65 antibodies (Cell Signaling Technology, USA) or nonspecific anti-IgG antibodies (negative control) (EMD Millipore, USA). After washing, the DNA was released, eluted, and PCR amplified, and the fragments were analyzed using agarose gel electrophoresis. The amount of precipitated DNA was calculated as the percentage of input sample. ChIP PCR primers were specific for the promoter regions containing putative p65 binding sites within miR-934; all primers used in this assay are summarized in Supplementary Table S1.

**Enzyme-linked immunosorbent assay (ELISA)**

To examine the effect of exosomes derived from HCT-8 cells on CXCL13 expression in PMA-treated THP-1 cells, ELISA was performed using a Human CXCL13 (BLC) ELISA Kit (Thermo Fisher Scientific, USA) according to the manufacturer’s instructions. The absorbance of the samples was measured at 490 nm using an ELISA plate reader within 30 min after the reaction was stopped.

**Supplementary Figure legends**

**Supplementary Figure S1.** **miR-934 is the top miRNA upregulated in CRLM compared to non-CRLM samples’** **primary tumor tissues.** qPCR analysis of the expression of the top ten upregulated miRNAs in 20 CRLM and 20 non-CRLM samples’primary tumor tissues from the FUSCC database.

**Supplementary Figure S2.** **Levels of miR-934 in the tissues and serum of CRC patients from the FUSCC dataset and the role of miR-934 in predicting the OS and DFS of CRC patients from the TCGA dataset. A**. Expression of miR-934 in 41 normal tissues and 110 CRC tissues. **B.** Expression of miR-934 in the serum of 41 healthy controls and 110 CRC patients. **C-D.** Kaplan-Meier survival analysis with the log-rank test was used to determine the association of miR-934 expression with the OS (**C**) and DFS (**D**) of CRC patients from the TCGA dataset (**p* < 0.05; ***p* < 0.01; ****p* < 0.001).

**Supplementary Figure S3. Spearman correlation analysis of the CD163 positivity rate and miR-934 expression in 50 CRC tissues.** Spearman correlation analysis showed that the CD163 positivity rate was positively associated with miR-934 expression in 50 CRC tissues.

**Supplementary Figure S4. Effects of exosomal miR-934 on the polarization of HBMDMs. A**. Morphology of human bone marrow-derived macrophages (HBMDMs). The macrophage marker CD11b was measured with flow cytometry. **B.** qPCR analysis of the changes in M2 marker (CD163, CD206, Arginase-1 and IL-10) expression levels after HBMDMs were treated with CRC cell-derived exosomes (**p* < 0.05; ***p* < 0.01; ****p* < 0.001).

**Supplementary Figure S5. Effect of miR-934 mimics and anti-miR-934 vectors on** **THP-1 cells prestimulated with PMA and CRC cells.**

**A.** Levels of miR-934 in THP-1 cells prestimulated with PMA and transfected with miR-934 mimics, anti-miR-934 vectors, or their control vectors. **B**. Levels of miR-934 in HT-29 and HCT8 cells transfected with miR-934 mimics, anti-miR-934 vectors, or their control vectors (***p* < 0.01; ****p* < 0.001).

**Supplementary Figure S6. Effect of the RNA binding proteins hnRNPU and hnRNPR on the expression of CRC cell-derived exosomal miR-934. A-B.** Changes in the mRNA and protein expression levels of hnRNPU and hnRNPR induced by transfection of their knockdown plasmids. **C-D.** Levels of total and exosomal miR-934 after transfection of shhnRNPU, shhnRNPR, or their negative control plasmids into HCT8 and LoVo cells (**p* < 0.05; ***p* < 0.01; ****p* < 0.001).

**Supplementary Figure S7.** Changes in the expression of PTEN induced by transfection of overexpression (**A**) or knockdown (**B**) vectors into PMA-treated THP-1 cells.

**Supplementary Figure S8. Representative HE staining images of each group (as a supplement to Fig. 6E).**

**Supplementary Figure S9. Changes in the secretion of CCL22 and IL-10 after THP-1 cells prestimulated with PMA were transfected with miR-934 mimics.** ELISA assays examined CCL22 (**A**) and IL-10 (**B**) in the CM of THP-1 cells prestimulated with PMA and transfected with miR-934 mimics (**p* < 0.05).

**Supplementary Figure S10. Effects of exosomal miR-934 on the secretion of CXCL13 by Kupffer cells.** ELISA measuring CXCL13 in the CM of human Kupffer cells pretreated with exosomes derived from HCT-8/HT-29 cells and transfected with anti-miR-934 or miR-934 mimics (**p* < 0.05; ***p* < 0.01).

**Supplementary Figure S11. Changes in the expression of CXCR5 induced by transfection of its knockdown vectors into SW480 cells.**

**Supplementary Figure S12. Representative HE staining images of each group (as a supplement to Fig. 7G).**

**Supplementary Figure S13. TAMs promote the migration and invasion of CRC cells by activating the CXCL13/CXCR5 axis. A-B.** TAMs were cocultured with CRC cells and anti-CXCL13 antibody or cocultured with CRC cells transfected with sh-CXCR5. Migration and invasion *in vitro* were evaluated using a transwell assay (*p < 0.05; **p < 0.01; ***p < 0.001).

**Supplementary Figure S14. Association of miR-934 expression in CRC tissues and paired liver metastatic tissues with CXCL13 and CXCR5 expression in** **CRC tissues, adjacent normal liver tissues and paired liver metastatic tissues. A.** A qPCR assay was used to examine miR-934 expression in 50 CRC tissues and paired liver metastatic tissues. **B.** Representative images of IHC staining of CXCL13 and CXCR5 in 50 CRC tissues, adjacent normal liver tissues and paired liver metastatic tissues (*p < 0.05; The red arrows indicate paired liver metastatic tissues; the black arrows indicate adjacent normal liver tissues; Scale bar, 200 μm.).
