# Supplementary material for: Tumor-derived exosomal miR-934 induces macrophage M2 polarization to promote liver metastasis of colorectal cancer
Source: J Hematol Oncol. 2020 Nov 19;13:156. doi: 10.1186/s13045-020-00991-2 (PMC7678301; doi:10.1186/s13045-020-00991-2)
Supplement: Supplementary file 16 — Additional file 16: Table S1. Data of sequences for qPCR and cell transfection in this study. [file 13045_2020_991_MOESM16_ESM.docx]

**Supplementary Table S1: Data of sequences for qPCR and cell transfection in this study.**

| **Gene** | **Sequence** |
| --- | --- |
| miR-934 | Forward: GCCTAGAAACATCCTCCCGG  Reverse: AGGCCATGTGTCGTGGTCG |
| CD68 | Forward: GGAAATGCCACGGTTCATCCA  Reverse: TGGGGTTCAGTACAGAGATGC |
| CD206 | Forward: GGGTTGCTATCACTCTCTATGC  Reverse: TTTCTTGTCTGTTGCCGTAGTT |
| Arginase-1 | Forward: GTGGAAACTTGCATGGACAAC  Reverse: AATCCTGGCACATCGGGAATC |
| IL-10 | Forward: CCTCCGTCTGTGTGGTTTGAA  Reverse: CACTGCGGTAAGGTCATAGGA |
| iNOS | Forward: GTTCCAGATGAATACTGGCAGTC  Reverse: GCAACTGAACACTATCTTTCCCT |
| IL-1β | Forward: ATGATGGCTTATTACAGTGGCAA  Reverse: GTCGGAGATTCGTAGCTGGA |
| CD163 | Forward: TTTGGACAAGCCGTGACTAGA  Reverse: CATTCCCGGTGTTGACATTCC |
| si-hnRNPA2B1-1 | Forward: GCGGAAUUAAAGAAGAUACTT  Reverse: GUAUCUUCUUUAAUUCCGCTT |
| si-hnRNPA2B1-2 | Forward: GCAAGACCUCAUUCAAUUGTT  Reverse: CAAUUGAAUGAGGUCUUGCTT |
| PTEN | Forward: TGGATTCGACTTAGACTTGACCT  Reverse: GGTGGGTTATGGTCTTCAAAAGG |
| sh-PTEN | Forward: GACCAACUCUCUCCUGUAUTT  Reverse: AUACAGGAGAGAGUUGGUCTT |
| LV-PTEN | Forward: GAGAAACTACTACCGACAGTACCACCGCTGGCCATGGGCCCCTAGGAG  Reverse: TCACCATGGTGGCGACCGGGACTTTTGTAATTTGTGAATG |
| MMP2 | Forward: TGACTTTCTTGGATCGGGTCG  Reverse: AAGCACCACATCAGATGACTG |
| MMP9 | Forward: GGGACGCAGACATCGTCATC  Reverse: TCGTCATCGTCGAAATGGGC |
| GAPDH | Forward: ACAACTTTGGTATCGTGGAAGG  Reverse: GCCATCACGCCACAGTTTC |
| U6 | Forward: GCTTCGGCAGCACATATACTAAAAT  Reverse: CGCTTCACGAATTTGCGTGTCAT |
| miR-934 promoter Region 1 | Forward: GGCATGAGATGAGGTGCCAA  Reverse: CGATATTACGCAGCAGCCCA |
| miR-934 promoter Region 2 | Forward: GCTCCAGGCAAACAACAAGG  Reverse: AACCCAAGGGAAACCAAGCA |
| miR-934 promoter Region 3 | Forward: TGTCATGTTTGGGAAACCTGTG  Reverse: GTCGAAACAGGGCTTTGCTG |
| miR-934 promoter Region 4 | Forward: TCAGGAACACTGCCTTGGAA  Reverse: CACATTGCCTGGCCCTAAGT |
| miR-934 promoter Region 5 | Forward: ACTACTGGAGACACTGGTAGTA  Reverse: TGAGTGATAGAAATAAGGCTCCCG |
