# Supplementary material for: Tumor-derived exosomal miR-934 induces macrophage M2 polarization to promote liver metastasis of colorectal cancer
Source: J Hematol Oncol. 2020 Nov 19;13:156. doi: 10.1186/s13045-020-00991-2 (PMC7678301; doi:10.1186/s13045-020-00991-2)
Supplement: Supplementary file 17 — Additional file 17: Table S2. Data of antibodies used in our research [file 13045_2020_991_MOESM17_ESM.docx]

**Supplementary Table S2:** Data of antibodies used in our research

| **Antibody** | **WB** | **IHC** | **RIP** | **ChIP** | **Specificity** | **Company** |
| --- | --- | --- | --- | --- | --- | --- |
| Tsg101 (ab125011) | 1:1000 | - | - | - | Rabbit monoclonal | Abcam |
| Aip1 (ab215049) | 1:1000 | - | - | - | Sheep polyclonal | Abcam |
| β1-Integrin (ab179471) | 1:1500 | - | - | - | Rabbit monoclonal | Abcam |
| Cd81 (ab109201) | 1:1000 | - | - | - | Rabbit monoclonal | Abcam |
| CD206  (ab125028) | 1:1000 | - | - | - | Rabbit monoclonal | Abcam |
| Arginase-1  (#93668) | 1:10000 | - |  | - | Rabbit monoclonal | Cell Signaling Technology |
| GAPDH (60004-1-lg) | 1:1000 | - | - | - | Mouse monoclonal | Proteintech |
| PTEN  (#9188) | 1:1000 | - | - | - | Rabbit monoclonal | Cell Signaling Technology |
| hnRNPA2B1 (ab6102) | 1:1500 | - | 1:50 | - | Mouse monoclonal | Abcam |
| Histone H3 (ab1791) | 1:1000 | - | - | - | Rabbit polyclonal | Abcam |
| Alix (ab117600) | 1:500 | - | - | - | Mouse monoclonal | Abcam |
| AKT  (#2920) | 1:2000 | - | - | - | Mouse monoclonal | Cell Signaling Technology |
| p-AKT  (#4060) | 1:2000 | - | - | - | Rabbit monoclonal | Cell Signaling Technology |
| PI3K  (#4292) | 1:1000 | - | - | - | Rabbit | Cell Signaling Technology |
| p-PI3K  (ab182651) | 1:800 | - | - | - | Rabbit polyclonal | Abcam |
| MMP2  (#87809) | 1:1000 | - | - | - | Rabbit monoclonal | Cell Signaling Technology |
| MMP9  (#13667) | 1:1000 | - | - | - | Rabbit monoclonal | Cell Signaling Technology |
| CXCR5  (ab203212) | 1:500 | 1:100 | - | - | Rabbit polyclonal | Abcam |
| p65  (#8242) | 1:1000 | - | - | 1:100 | Rabbit monoclonal | Cell Signaling Technology |
| p-p65  (#3033) | 1:1000 | - | - | - | Rabbit monoclonal | Cell Signaling Technology |
| IκBα  (ab32518) | 1:1000 | - | - | - | Rabbit monoclonal | Abcam |
