# Supplementary material for: Tumor-derived exosomal miR-934 induces macrophage M2 polarization to promote liver metastasis of colorectal cancer
Source: J Hematol Oncol. 2020 Nov 19;13:156. doi: 10.1186/s13045-020-00991-2 (PMC7678301; doi:10.1186/s13045-020-00991-2)
Supplement: Supplementary file 19 — Additional file 19: Table S4. Expression of miR-934 in normal colorectal mucosa and primary cancerous tissues (n = 308) [file 13045_2020_991_MOESM19_ESM.docx]

**Supplementary Table S4:** **Expression of miR-934 in normal colorectal mucosa and primary cancerous tissues (n=308).**

| Tissue sample | Expression of miR-934 | | | | | *P* value | |
| --- | --- | --- | --- | --- | --- | --- | --- |
|  | | Low expression (n, %) | | High expression (n, %) | | |  |
| Normal mucosa | 223 (72.4) | | 85 (27.6) | | <0.001* | | |
| Tumor tissue | 123 (39.9) | | 185 (60.1) | |  | | |

* The significant difference in the expression of miR-934 between normal colorectal mucosa and cancerous tissues
