# Supplementary material for: Tumor-derived exosomal miR-934 induces macrophage M2 polarization to promote liver metastasis of colorectal cancer
Source: J Hematol Oncol. 2020 Nov 19;13:156. doi: 10.1186/s13045-020-00991-2 (PMC7678301; doi:10.1186/s13045-020-00991-2)
Supplement: Supplementary file 20 — Additional file 20: Table S5. Associations between miR-934 expression and clinicopathological characteristics in 308 CRC patients. [file 13045_2020_991_MOESM20_ESM.docx]

**Supplementary Table S5: Associations between miR-934 expression and clinicopathological characteristics in 308 CRC patients.**

| Variable | N | miR-934 expression | | *P* value |
| --- | --- | --- | --- | --- |
|  | 308 | Low expression (123) | High expression (185) |  |
| Age (yr) |  |  |  | 0.946 |
| <57 | 142 | 57 (40.1%) | 85 (59.9%) |  |
| >=57 | 166 | 66 (39.8%) | 100 (60.2%) |  |
| Gender |  |  |  | 0.432 |
| Male | 182 | 76 (41.8%) | 106 (58.2%) |  |
| Female | 126 | 47 (37.3%) | 79 (62.7%) |  |
| Tumor location |  |  |  | 0.045* |
| Right | 65 | 34 (52.3%) | 31 (47.7%) |  |
| Transverse | 6 | 1 (16.7%) | 5 (83.3%) |  |
| Left | 16 | 3 (18.8%) | 13 (81.3%) |  |
| Sigmoid  Rectum | 59  162 | 26 (44.1%)  59 (36.4%) | 33 (55.9%)  103 (63.6%) |  |
| T classification |  |  |  | <0.001* |
| Tx | 1 | 1 (100.0%) | 0 (0.0%) |  |
| T 1 | 2 | 2 (100.0%) | 0 (0.0%) |  |
| T 2 | 45 | 28 (62.2%) | 17 (37.8%) |  |
| T 3 | 53 | 26 (49.1%) | 27 (50.9%) |  |
| T 4 | 207 | 66 (31.9%) | 141 (68.1%) |  |
| N classification |  |  |  | 0.542 |
| N 0 | 129 | 56 (43.4%) | 73 (56.6%) |  |
| N 1 | 96 | 37 (38.5%) | 59 (61.5%) |  |
| N 2 | 83 | 30 (36.1%) | 53 (63.9%) |  |
| M classification |  |  |  | <0.001* |
| M 0 | 249 | 115 (46.2%) | 134 (53.8%) |  |
| M 1 | 59 | 8 (13.6%) | 51 (86.4%) |  |
| AJCC stage |  |  |  | <0.001* |
| I | 27 | 18 (66.7%) | 9 (33.3%) |  |
| II | 90 | 34 (37.8%) | 56 (62.2%) |  |
| III | 132 | 63 (47.7%) | 69 (52.3%) |  |
| IV | 59 | 8 (13.6%) | 51 (86.4%) |  |
| Differentiation |  |  |  | 0.646 |
| Well | 16 | 8 (50.0%) | 8 (50.0%) |  |
| Moderate | 217 | 84 (38.7%) | 133 (61.3%) |  |
| Poor | 75 | 31 (41.3%) | 44 (58.7%) |  |
| Recurrence |  |  |  | 0.002* |
| No | 253 | 111 (43.9%) | 142 (56.1%) |  |
| Yes | 55 | 12 (21.8%) | 43 (78.2%) |  |

*P<0.05 indicates significant difference
