# Supplementary material for: Tumor-derived exosomal miR-934 induces macrophage M2 polarization to promote liver metastasis of colorectal cancer
Source: J Hematol Oncol. 2020 Nov 19;13:156. doi: 10.1186/s13045-020-00991-2 (PMC7678301; doi:10.1186/s13045-020-00991-2)
Supplement: Supplementary file 21 — Additional file 21: Table S6. Univariate and multivariate analysis of overall survival in 308 CRC patients [file 13045_2020_991_MOESM21_ESM.docx]

**Supplementary Table S6: Univariate and multivariate analysis of overall survival in 308 CRC patients.**

|  | Univariate analysis | | Multivariate analysis | |
| --- | --- | --- | --- | --- |
|  | HR (95%CI) | *P* Value | HR (95%CI) | *P* Value |
| Age (yr) | 1.117 (0.726-1.718) | 0.616 |  |  |
| Gender | 0.993 (0.643-1.532) | 0.974 |  |  |
| Tumor location | 1.063 (0.932-1.212) | 0.361 |  |  |
| T classification | 2.850 (1.755-4.630) | <0.001* | 1.828 (1.105-3.028) | 0.019* |
| N classification | 2.360 (1.793-3.106) | <0.001* | 1.284 (0.902-1.826) | 0.165 |
| M classification | 10.753 (6.892-16.777) | <0.001* | 5.364 (3.254-8.844) | <0.001* |
| AJCC stage  (III-IV vs I-II) | 9.126 (4.205-19.807) | <0.001* | 2.988 (1.126-7.930) | 0.028* |
| Differentiation | 0.732 (0.485-1.104) | 0.137 |  |  |
| Recurrence | 2.693 (1.705-4.253) | <0.001* | 2.431 (1.504-3.930) | <0.001* |
| miR-934 | 4.641 (2.609-8.257) | <0.001* | 2.740 (1.487-5.051) | 0.001* |

HR hazard ratio; CI confidence interval

* *P*<0.05 indicate that the 95% CI of HR was not including 1
