# Supplementary material for: Tumor-derived exosomal miR-934 induces macrophage M2 polarization to promote liver metastasis of colorectal cancer
Source: J Hematol Oncol. 2020 Nov 19;13:156. doi: 10.1186/s13045-020-00991-2 (PMC7678301; doi:10.1186/s13045-020-00991-2)
Supplement: Supplementary file 22 — Additional file 22: Table S7. Univariate and multivariate analysis of disease-free survival in 308 CRC patients. [file 13045_2020_991_MOESM22_ESM.docx]

**Supplementary Table S7: Univariate and multivariate analysis of disease-free survival in 308 CRC patients.**

|  | Univariate analysis | | Multivariate analysis | |
| --- | --- | --- | --- | --- |
|  | HR (95%CI) | *P* Value | HR (95%CI) | *P* Value |
| Age (yr) | 0.914 (0.516-1.619) | 0.757 |  |  |
| Gender | 1.209 (0.682-2.144) | 0.516 |  |  |
| Tumor location | 0.968 (0.802-1.168) | 0.733 |  |  |
| T classification | 2.616 (1.455-4.702) | 0.001* | 2.086 (1.070-4.068) | 0.031* |
| N classification | 2.139 (1.500-3.052) | <0.001* | 0.962 (0.561-1.647) | 0.962 |
| M classification | 7.238 (3.654-14.337) | <0.001* | 5.918 (2.773-12.629) | <0.001* |
| AJCC stage  (III-IV vs I-II) | 5.776 (2.585-12.906) | <0.001* | 3.687 (1.212-11.213) | 0.022* |
| Differentiation | 0.813 (0.464-1.424) | 0.469 |  |  |
| Recurrence | 8.733 (4.828-15.795) | <0.001* | 6.396 (3.317-12.332) | <0.001* |
| miR-934 | 4.695 (2.266-9.729) | <0.001* | 3.656 (1.717-7.786) | 0.001* |

HR hazard ratio; CI confidence interval

* *P*<0.05 indicates that the 95% CI of HR was not including 1
