# Supplementary material for: Tumor-derived exosomal miR-934 induces macrophage M2 polarization to promote liver metastasis of colorectal cancer
Source: J Hematol Oncol. 2020 Nov 19;13:156. doi: 10.1186/s13045-020-00991-2 (PMC7678301; doi:10.1186/s13045-020-00991-2)
Supplement: Supplementary file 24 — Additional file 24: Table S9. Association of miR-934 expression with CXCL13 and CXCR5 expression in CRC tissues (n = 50). Table S10: Association of miR-934 expression with CXCL13 and CXCR5 expression in paired liver metastatic tissues (n = 50). [file 13045_2020_991_MOESM24_ESM.docx]

**Supplementary Table S9: Association of miR-934 expression with CXCL13 and CXCR5 expression in CRC tissues (n=50).**

| CRC Tissue | Expression of miR-934 | | | | | *P* value | |
| --- | --- | --- | --- | --- | --- | --- | --- |
|  | | Low expression (n, %) | | High expression (n, %) | | |  |
| CXCL13 Low | 13(65.0) | | 8(26.7) | | 0.01* | | |
| CXCL13 High | 7(35.0) | | 22(73.3) | |  | | |
| CXCR5 Low | 11 (72.4) | | 7 (27.6) | | 0.035* | | |
| CXCR5 High | 9 (39.9) | | 23 (60.1) | |  | | |

**P* <0.05 indicates significance

**Supplementary Table S10:** **Association of miR-934 expression with CXCL13 and CXCR5 expression in paired liver metastatic tissues (n=50).**

| Liver Metastatic Tissue | Expression of miR-934 | | | | | *P* value | |
| --- | --- | --- | --- | --- | --- | --- | --- |
|  | | Low expression (n, %) | | High expression (n, %) | | |  |
| CXCL13 Low | 10(83.3) | | 10(26.3) | | 0.001* | | |
| CXCL13 High | 2(16.7) | | 28(76.7) | |  | | |
| CXCR5 Low | 8 (72.4) | | 8 (27.6) | | 0.01* | | |
| CXCR5 High | 4 (39.9) | | 30 (60.1) | |  | | |

**P* <0.05 indicates significance
