# Supplementary material for: Tumor-derived exosomal miR-934 induces macrophage M2 polarization to promote liver metastasis of colorectal cancer
Source: J Hematol Oncol. 2020 Nov 19;13:156. doi: 10.1186/s13045-020-00991-2 (PMC7678301; doi:10.1186/s13045-020-00991-2)
Supplement: Supplementary file 25 — Additional file 25: Table S11. The wild (WT) and mutant (MUT) sequences of binding sites between p65 and the promoter of miR-934. [file 13045_2020_991_MOESM25_ESM.docx]

**Supplementary Table S11: The wild (WT) and mutant (MUT) sequences of binding sites between p65 and the promoter of miR-934.**

|  | **Stat** | **End** | **WT** | **MUT** |
| --- | --- | --- | --- | --- |
| 1 | -152 | -143 | TAGAAATTTC | TAATTATAAC |
| 2 | -88 | -79 | TGGATCTGCC | TAAATCAGCC |
